# Loss of the DYRK1A Protein Kinase Results in the Reduction in Ribosomal Protein Gene Expression, Ribosome Mass and Reduced Translation

**DOI:** 10.3390/biom14010031

**Published:** 2023-12-25

**Authors:** Chiara Di Vona, Laura Barba, Roberto Ferrari, Susana de la Luna

**Affiliations:** 1 Centre for Genomic Regulation (CRG), The Barcelona Institute of Science and Technology (BIST), Dr Aiguader 88, 08003 Barcelona, Spain; 2 Centro de Investigación Biomédica en Red en Enfermedades Raras (CIBERER), 28029 Madrid, Spain; 3 Department of Chemistry, Life Sciences and Environmental Sustainability, University of Parma, Viale delle Scienze 23/A, 43124 Parma, Italy; roberto.ferrari1@unipr.it; 4 Department of Medicine and Life Sciences, Universitat Pompeu Fabra (UPF), Dr Aiguader 88, 08003 Barcelona, Spain; 5 Institució Catalana de Recerca i Estudis Avançats (ICREA), Passeig Lluís Companys 23, 08010 Barcelona, Spain

**Keywords:** ribosomal proteins, TCTCGCGAGA, translation, DYRK1A, transcription

## Abstract

Ribosomal proteins (RPs) are evolutionary conserved proteins that are essential for protein translation. RP expression must be tightly regulated to ensure the appropriate assembly of ribosomes and to respond to the growth demands of cells. The elements regulating the transcription of RP genes (RPGs) have been characterized in yeast and *Drosophila*, yet how cells regulate the production of RPs in mammals is less well understood. Here, we show that a subset of RPG promoters is characterized by the presence of the palindromic TCTCGCGAGA motif and marked by the recruitment of the protein kinase DYRK1A. The presence of DYRK1A at these promoters is associated with the enhanced binding of the TATA-binding protein, TBP, and it is negatively correlated with the binding of the GABP transcription factor, establishing at least two clusters of RPGs that could be coordinately regulated. However, DYRK1A silencing leads to a global reduction in RPGs mRNAs, pointing at DYRK1A activities beyond those dependent on its chromatin association. Significantly, cells in which DYRK1A is depleted have reduced RP levels, fewer ribosomes, reduced global protein synthesis and a smaller size. We therefore propose a novel role for DYRK1A in coordinating the expression of genes encoding RPs, thereby controlling cell growth in mammals.

## 1. Introduction

Ribosomes are cellular machines that translate mRNA into protein, and in mammals, they are formed by the large 60S subunit and the small 40S subunit. The 60S subunit is comprised of the 5S, 5.8S and 28S rRNAs associated with 52 ribosomal proteins (RPs), and the smaller 40S subunit is made up of the 18S rRNA plus 35 RPs. Ribosome biogenesis is a complex process that involves more than 200 different factors: rRNAs, small nucleolar RNAs and canonical and auxiliary RPs [1]. The three RNA polymerases (Pol) participate in the transcription of the ribosomal components, with Pol I responsible for transcribing the 28S, 18S and 5.8S rRNAs, Pol III transcribing the 5S rRNAs and Pol II responsible for the transcription of all the protein coding genes involved in ribosome biogenesis, including the RP genes (RPGs). Therefore, the coordinated expression of these components is required to ensure the correct assembly and proper functioning of ribosomes [2]. Indeed, the dysregulation of ribosome biogenesis is associated with a group of human diseases that are collectively known as ribosomopathies [3]; moreover, alterations to RP expression contribute to cancer cell growth [4].

The coding sequences of RPGs have been highly conserved over evolution, unlike the features of their promoters and the machinery involved in their transcriptional regulation. As such, RPGs are organized into operons in prokaryotes [5], whereas the situation is much more complex in the case of eukaryotes, with multiple genes widely scattered across the genome [6]. The main elements involved in the transcriptional regulation of RPGs have been characterized thoroughly in *Saccharomyces cerevisiae* [7], in which the repressor activator protein 1 (Rap1p) and the Fhl1p forkhead transcription factor (TF) are constitutively bound to the RPG promoters, coordinating RPG expression [8]. In higher eukaryotes, most studies have focused on the differential enrichment of TF binding motifs within RPG promoters [9,10,11,12]. In particular, several DNA sequences are found over-represented in human RPG promoters. The polypyrimidine TCT motif is found close to the transcription start site (TSS) of RPGs, and it is thought to play a dual role in the initiation of both transcription and translation [13]. This motif is recognized by the TATA-box-binding protein (TBP)-related factor 2 (TRF2) in *Drosophila* [14,15], yet it remains unclear whether there is functional conservation with its human TBP-like 1 (TBPL1) homolog. Around 35% of the RPG promoters contain a TATA box in the -25 region and an additional 25% contain A/T-rich sequences in this region [16]. Other motifs frequently found are those for SP1, the GA-binding protein (GABP) and the yin yang 1 (YY1) TFs [16]. In addition, the E-box TF MYC is a key regulator of ribosomal biogenesis, enhancing the expression of RPGs [17]. Finally, a de novo motif (M4 motif) was found enriched in human and mouse RPG promoters [9]. RPG mRNA expression displays tissue- and development-specific patterns, both in human and mouse [6,18,19]. Hence, RPG expression could be regulated by specific combinations of TFs in different organisms and/or physiological conditions.

The M4 motif matches the palindromic sequence that is bound by the dual-specificity tyrosine-regulated kinase 1A (DYRK1A) protein kinase [20]. DYRK1A fulfills many diverse functions by phosphorylating a wide range of substrates [21,22,23], and it is a kinase with exquisite gene-dosage dependency. On the one hand, DYRK1A overexpression in individuals with trisomy 21 has been associated to several of the pathological symptoms associated with Down syndrome (DS) [24]. On the other hand, de novo mutations in one *DYRK1A* allele cause a rare clinical syndrome known as DYRK1A haploinsufficiency syndrome (OMIM#614104) [25,26,27]. DYRK1A has also been proposed as a pharmacological target for neurodegenerative disorders, diabetes and cancer [22,23,28,29]. We have shown that DYRK1A is a transcriptional activator when recruited to proximal promoter regions of a subset of genes that are enriched for the palindromic motif TCTCGCGAGA [20]. DYRK1A phosphorylates serine residues 2, 5 and 7 within the C-terminal domain (CTD) of the catalytic subunit of Pol II [20]. This activity takes over that of the general TF p-TEFb at gene loci involved in myogenic differentiation [30]. The interaction of DYRK1A with the CTD depends on a run of histidine residues in its noncatalytic C-terminus, which also promotes the nucleation of a phase-separated compartment that is functionally associated with transcriptional elongation [31]. Here, we have analyzed the occupancy of RPG promoters by DYRK1A in depth, performing a comprehensive analysis of the promoter occupancy by other factors whose binding motifs are differentially enriched in human RPG promoter regions. Our results indicate that most of these factors are found at almost all RPG promoters, irrespective of the presence of their cognate binding sites. By contrast, DYRK1A associates with a subset of human and mouse RPG promoters that contain the TCTCGCGAGA motif. Moreover, physiological levels of DYRK1A are required to maintain RPG transcript levels independently of the binding of DYRK1A to their promoters, and this effect could at least in part contribute to the global reduction in ribosome mass and protein synthesis when DYRK1A is silenced. Therefore, our results expand the functional spectrum of the DYRK1A kinase, indicating that it contributes to the regulation of cell growth in mammalian cells.

## 2. Materials and Methods

### 2.1. Cell Culture and Lentivirus-Mediated Transduction

Lentiviral transduction of short hairpin (sh)RNAs was used to downregulate DYRK1A expression, and the generation of the lentiviral stocks and the infection conditions are detailed in the Supplementary Methods. The protocols to determine the cell cycle profile and cell volume are also included in the Supplementary Methods. To analyze global protein synthesis, T98G cells were incubated for 90 min in methionine-free Dulbecco’s modified Eagle’s medium (DMEM; GIBCO, Waltham, MA, USA) with 10% dialyzed fetal bovine serum (FBS; GIBCO), metabolically labeled for 20 min with ^35^S-Met (50 μCi ^35^S-Met, 1175 Ci/mmol, Perkin Elmer) and then lysed in SDS lysis buffer. The protein extracts were resolved by SDS-PAGE and the incorporation of ^35^S-methionine was detected by the autoradiography of the dried gel using film or a Phosphoimager (Typhoon Trio, GE Healthcare, Chicago, IL, USA).

### 2.2. Preparation of Polysome and Ribosome-Enriched Fractions

Polysome profiles were obtained from approximately 1 × 10^7^ T98G cells. Protein synthesis was arrested by incubation with cycloheximide (CHX, 100 μg/mL). The cells were washed in phosphate-buffered saline (PBS) containing CHX (100 μg/mL), collected in 1 mL of polysome lysis buffer (10 mM Tris-HCl with a pH of 7.4, 100 mM NaCl, 10 mM MgCl_2_, 1% Triton X-100, 20 mM dithiothreitol [DTT], 100 μg/mL CHX, 0.25% sodium deoxycholate) and frozen rapidly in liquid nitrogen. Cell debris and nuclei were eliminated by centrifugation (12,000× *g*, 5 min, 4 °C) and the nucleic acid concentration in the supernatants was assessed by measuring the A_260_ in a NanoDrop™ (Thermo Fisher Inc, Waltham, MA, USA). Samples with A_260_ ≈ 10 were loaded onto a 10–50% linear sucrose gradient prepared in polysome gradient buffer (20 mM Tris-HCl with a pH of 7.4, 100 mM NH_4_Cl, 10 mM MgCl_2_, 0.5 mM DTT, 100 μg/mL CHX) and centrifuged in a Beckman SW41Ti rotor (35,000 rpm, 3 h, 4 °C). Profiles were obtained by continuous monitoring of the A_254_ (Econo-UV Monitor and Econo-Recorder model 1327; Bio-Rad Laboratories, Hercules, CA, USA). To calculate the polysome:monosome ratio, the polysome and monosome area under the curve was measured with ImageJ (1.50i) [32].

To isolate the total ribosome fraction, cells were collected in sucrose buffer (250 mM sucrose, 250 mM KCl, 5 mM MgCl_2_, 50 mM Tris-HCl with a pH of 7.4, 0.7% Nonidet P-40), the cytosol was isolated by centrifugation (750× *g*, 10 min, 4 °C) and then centrifuged again to obtain a postmitochondrial supernatant (12,500× *g*, 10 min, 4 °C). The supernatant was adjusted to 0.5 M KCl, and the volume equivalent to OD_260_ = 5 was loaded onto a sucrose cushion (1 M sucrose, 0.5 M KCl, 5 mM MgCl_2_, 50 mM Tris-HCl with a pH of 7.4) and centrifuged in a Beckman TLA 100.3 rotor (250,000× *g*, 2 h, 4 °C).

### 2.3. Mass Spectrometry (MS) Analysis

Proteins in the ribosome-enriched pellets were identified and quantified by free-label MS analysis using an LTQ-Orbitrap Fusion Lumos (Thermo Fisher Inc) mass spectrometer. The sample preparation, chromatography and MS analysis are detailed in the Supplementary Methods. For the peptide identification, a precursor ion mass tolerance of 7 ppm was used for MS1, with trypsin as the chosen enzyme and up to three miscleavages allowed. The fragment ion mass tolerance was set to 0.5 Da for MS2. The oxidation of methionine and N-terminal protein acetylation were used as variable modifications, whereas carbamidomethylation on cysteine was set as a fixed modification. In the analysis of phosphorylated peptides, phosphorylation of serine, threonine and tyrosine were also set as variable modifications. The false discovery rate (FDR) was set to a maximum of 5% in the peptide identification. Protein quantification was retrieved from the protein TOP3 Area node from Proteome Discoverer (v2.3). For normalization, a correction factor was applied: sum TOP3 replicate “n”/average sum TOP3 all replicates. Normalized abundance values were log_2_-transformed, and the fold change (FC) and *p*-values were calculated. Two independent experiments, each with three biological replicates, were performed on T98G cells transduced with shControl or shDYRK1A lentiviruses, and only those proteins detected in at least three replicates of any condition were quantified. For the RP stoichiometry, the intensity of each RP was defined relative to the intensities of all RPs. The RP protein/mRNA ratios were obtained using the log_2_-transformed normalized protein abundance and the log_2_-transformed normalized RNA counts from RNA-Seq experiments.

### 2.4. Chromatin Immunoprecipitation (ChIP)

Detailed information on sample preparation is provided in the Supplementary Methods. DNA libraries were generated with the Ovation^®^ Ultralow Library System V2 (NuGEN Technologies, San Carlos, CA, USA). Libraries were sequenced with 50 bp single-end reads on an Illumina Hiseq-2500 sequencer at the CRG Genomics Unit. The ChIP-Seq analysis was performed as described [33], with few modifications (see Supplementary Methods). To analyze the RPG promoter occupancy, datasets from The Encyclopedia of DNA Elements (ENCODE) Consortium were used and are listed in Table S2 [34]. Read numbers were normalized to reads per million (RPM) in both this work and the ENCODE datasets.

### 2.5. RNA-Seq

RNA was isolated with the RNeasy extraction kit (Qiagen, Germantown, MD, USA) and the samples were treated with DNase I (see Supplementary Methods for full details). For T98G cell spike-in normalization, equal numbers of T98G cells for each condition were mixed with a fixed number of *D. melanogaster* Kc167 cells (1:4 ratio). Libraries were prepared with the TruSeq Stranded mRNA Sample Prep Kit v2 (Illumina, Cambridge, UK) and sequenced with Illumina Hiseq-2500 to obtain 125 bp pair-ended reads. Differential gene expression was assessed with the DESeq2 (1.30.1) package in R, filtering genes that had >10 average normalized counts per million [35]. For the spike-in libraries, the size factor of each replicate was calculated according to exogenous *Drosophila* spike-in reads. Expression was considered to be altered when the *p*-value ≤ 0.05, and the log_2_FC was above 0.7 and below −0.7 for up- and downregulated genes, respectively.

### 2.6. Quantitative PCR (qPCR)

PCR reactions were performed in triplicate in 384-well plates with SYBR Green (Roche, Basel, Switzerland) and specific primers using a Roche LC-480 machine. The crossing point was calculated for each sample with the Lightcycler 480 1.2 software. No PCR products were observed in the absence of template and all primer sets gave narrow single melting point curves. For the ChIP-qPCR, a 1/10 dilution of ChIP DNA was used as the template for the PCR reaction, and a 1/1000 dilution of input DNA was used as the standard for normalization. For the RT-qPCR, a 1/10 dilution of the cDNAs was used and expression of the *D. melanogaster* gene *Act42A* was used for normalization. The sequences of primers are listed in Tables S3 and S4.

### 2.7. Computational Tools and Statistical Analysis

Full details for the computational tools used to analyze the ChIP-Seq, RNA-Seq and proteomics data are included in the Supplementary Methods. To calculate the statistical significance, the normality of the samples was evaluated with the Shapiro–Wilk normality test (Prism 5, v5.0d), and parametric or nonparametric tests were used accordingly. Statistical significance was calculated with two-tailed Mann–Whitney or Student’s tests for unpaired samples or with a Wilcoxon matched-pairs signed-ranks test (Prism 5, v5.0d), and a *p*-value ≤ 0.05 was considered as significant. All experiments were performed independently at least three times.

## 3. Results

### 3.1. DYRK1A Is Recruited to the Proximal Promoter Regions of the Canonical RPGs

**Figure 1:**
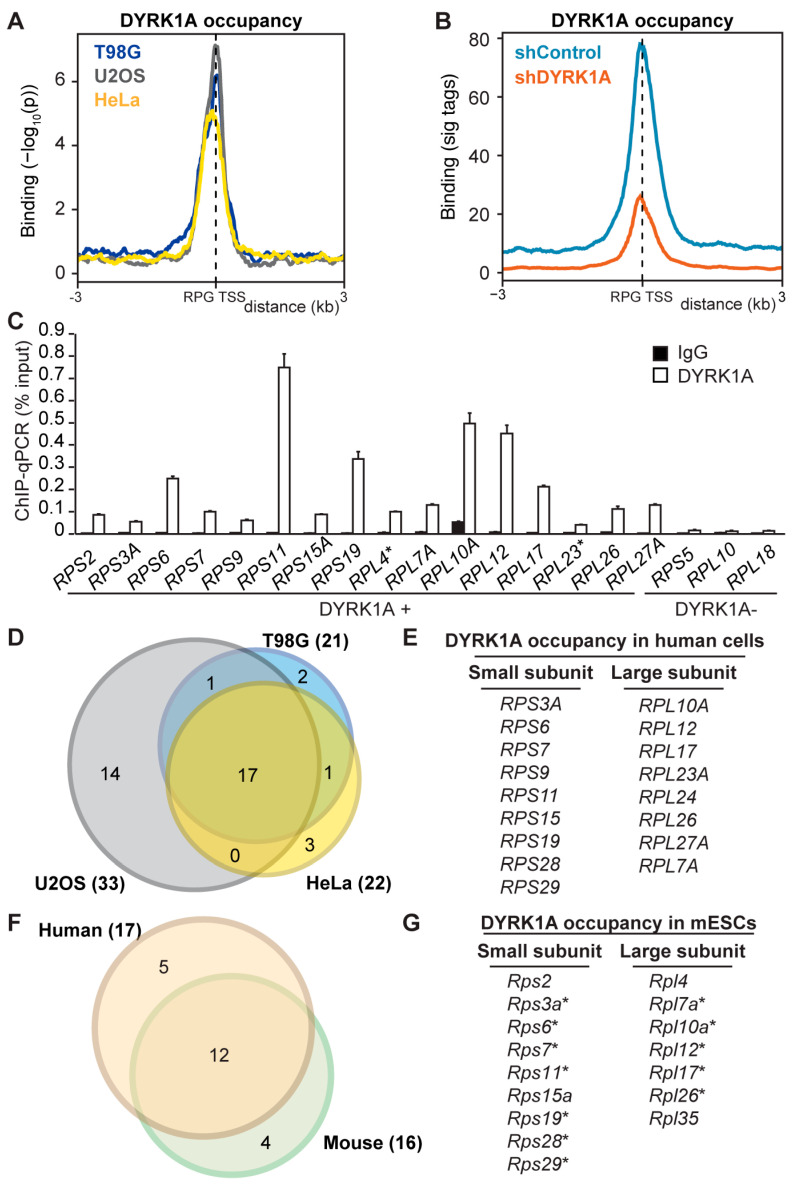
DYRK1A occupies a subset of RPG promoters. (**A**) Distribution of chromatin-bound DYRK1A relative to the TSS of human RPGs in T98G, U2OS and HeLa cells. The *y*-axis represents the relative protein recruitment (−log_10_ Poisson *p*-value) and the offset was set to ±3 kb from the TSS. (**B**) DYRK1A occupancy relative to the TSS of human RPGs, comparing the shControl and shDYRK1A T98G cells (blue and orange lines, respectively). The *y*-axis represents the relative protein recruitment quantified as significant (sig) ChIP-Seq tags. The offset was set to ±3 kb from the TSS (Figure S1B for representative examples). (**C**) Validation of the selected targets by ChIP-qPCR (percentage of input recovery; mean ± SD of three technical replicates). (**D**) Overlap of DYRK1A-associated RPG promoters in T98G, U2OS and HeLa cells. (**E**) List of DYRK1A-positive RPGs common to the three human cell lines. (**F**) Overlap between common DYRK1A-bound RPGs in the human cell lines and mESCs. (**G**) List of RPGs with DYRK1A detected at their promoters in mESCs. The asterisk indicates the coincident occupancy in mESCs with the three human cell lines analyzed.

Our prior analysis of DYRK1A recruitment to chromatin showed an enrichment in gene-ontology terms related to ribosome biogenesis and translational regulation [20]. In addition, a de novo motif analysis found a sequence similar to the DYRK1A-associated motif to be over-represented in the promoter regions of human RPGs [9]. Accordingly, we examined whether DYRK1A was recruited to human RPGs, considering the genes encoding the 80 canonical RPs plus 10 paralogues [6] (a new naming system for RPs has been proposed [36] and listed in Table S5). In the experiments on different human cell lines, the ChIP-Seq analysis showed DYRK1A bound upstream of the TSS and, in general, within 500 bp of the TSS of a subset of the RPGs (Figure 1A). No ChIP signal was detected at either the gene bodies or the transcriptional termination sites (TTS; Figure S1). A clear reduction in the chromatin-associated DYRK1A was observed at the target RPG promoters in cells where the levels of DYRK1A were depleted by the lentiviral delivery of a shRNA targeting DYRK1A, reflecting the specific recruitment of the kinase (Figure 1B and Figure S1). Finally, the presence of DYRK1A at the RPG promoter regions was further confirmed in independent ChIP-qPCR experiments (Figure 1C).

Around 25% of the promoters of all the RPGs were occupied by DYRK1A in the human cell lines analyzed, with considerable overlap among them (Figure 1D,E). No association with any particular ribosomal subunit was observed, since DYRK1A occupancy was detected similarly at the promoters of RPGs from both the large- and small-ribosome subunits (Figure 1E). Furthermore, the DYRK1A ChIP-Seq data from the mESCs also showed the presence of this kinase at the promoters of a subset of mouse RPGs, which coincided well with the DYRK1A-positive subset in the human cells (Figure 1F,G). Together, these results indicate that DYRK1A is recruited to proximal promoter regions of a subset of RPGs in different human and mouse cell lines, suggesting that the chromatin association of DYRK1A with these RPGs represents a general and conserved function for the kinase.

### 3.2. The TCTCGCGAGA Motif Marks the Subset of the RPG Promoters Positive for DYRK1A

**Figure 2:**
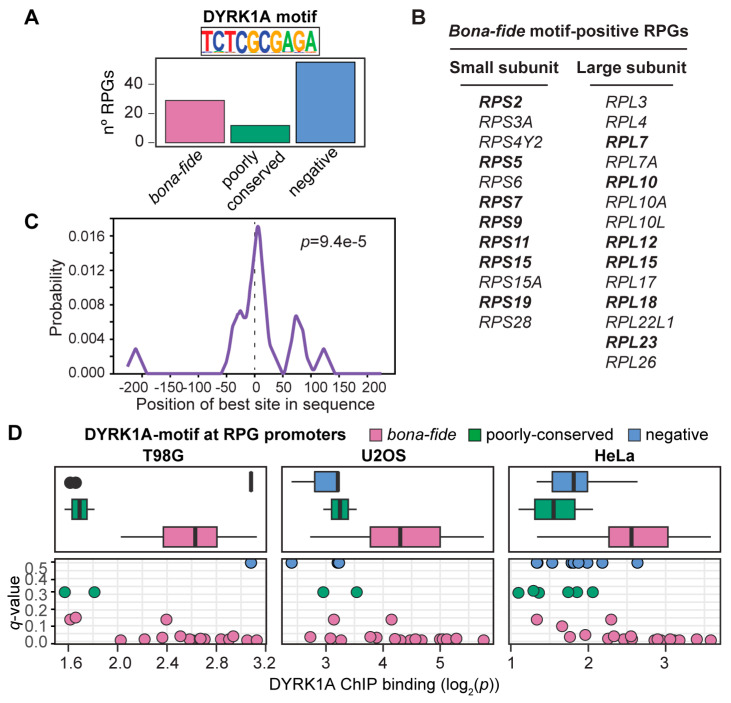
The TCTCGCGAGA motif correlates with significant DYRK1A binding at human RPG promoters. (**A**) Distribution of the TCTCGCGAGA motif (DYRK1A motif) in human RPGs (bona fide: *p*-value < 10^−4^; poorly conserved: 10^−4^ < *p*-value < 3 × 10^−4^). (**B**) List of human RPGs with the bona fide palindromic motif in their promoters. The RPG promoters containing more than one DYRK1A motif are in bold. (**C**) CentriMo plot showing the distribution of the DYRK1A motif for the RPG promoters that bind DYRK1A. The solid curve shows the positional distribution (averaged over bins of a 10 bp width) of the best site of the DYRK1A motif at each position in the RPG-ChIP-Seq peaks (500 bp). The *p*-value is for the central enrichment of the motif. (**D**) Box plot and scatter plots (dots represent the individual RPGs) showing the correlation between DYRK1A ChIP binding (log_2_ *p*-value, *x*-axis) and the conservation of the DYRK1A motif (*q*-value, *y*-axis) in T98G, U2OS and HeLa cells. The colors represent the motif-classification indicated.

Around 25% of the human RPG promoters contain a TCTCGCGAGA motif (Figure 2A,B), and DYRK1A bound to these in one, two or in all the human cell lines analyzed, except for *RPL10* and *RPS5*, and the RPG paralogs *RPL10L*, *RPL22L1* and *RPS4Y2* (Table S5). Likewise, DYRK1A was almost exclusively detected at RPG promoters containing the TCTCGCGAGA motif in the mESCs (Table S6), as further evidence of its functional conservation. A central model motif analysis (CMEA) [37] showed the TCTCGCGAGA motif to be positioned precisely around the center of the DYRK1A-associated peaks within the RPG promoters (Figure 2C), suggesting that it serves as a platform for DYRK1A recruitment. The kinase was also found to associate with a small number of RPG promoters without this palindromic sequence (Table S5), although its detection was poorer in these cases (Figure 2D). In conclusion, the TCTCGCGAGA motif appears to be a major determinant for the efficient recruitment of DYRK1A to the RPGs; moreover, given that the variability in DYRK1A occupancy in the different cell lines was mostly restricted to the RPG promoters without the motif, it is possible that DYRK1A associates with promoters containing this motif independently of the cell type, whereas the occupancy of other RPG promoters might be context-specific.

The TCTCGCGAGA motif has been shown to work as a promoter element that drives bidirectional transcription [38]. In around 30% of the RPGs, the TSS lies within a 500 bp window from the TSS of the genes transcribed in the opposite direction; in these cases, the RPG is always transcribed more strongly (Table S7). However, no specific bias for the presence of the TCTCGCGAGA motif could be observed, since some RPGs with bidirectional transcription contained this motif (*RPL12*, *RPL15*, *RPL23A*), while others did not (*RPL34*, *RPL9*, *RPS18*; Table S7). The transcriptional repressor zinc finger and BTB domain containing 33 (ZBTB33, also known as KAISO) binds directly to the TCTCGCGAGA motif in vitro when methylated [39]. Indeed, this palindromic motif is included in the Jaspar database of the curated TF binding profiles as a ZBTB33 motif (http://jaspar.genereg.net/matrix/MA0527.1/ (accessed on 1 January 2021)). The ChIP-Seq experiments in the T98G cells did detect ZBTB33 at the majority of the RPG promoters containing the TCTCGCGAGA motif (Figure S2A; Table S8), with signals overlapping those of DYRK1A (Figure S2B). Moreover, the TCTCGCGAGA motif is positioned centrally within the ZBTB33-bound regions of these RPGs (Figure S2C). Thus, this promoter motif might not only favor DYRK1A recruitment, but also its interaction with other proteins.

### 3.3. Low Functional Conservation of the RPG Core Promoter Elements between Drosophila and Humans

**Figure 3:**
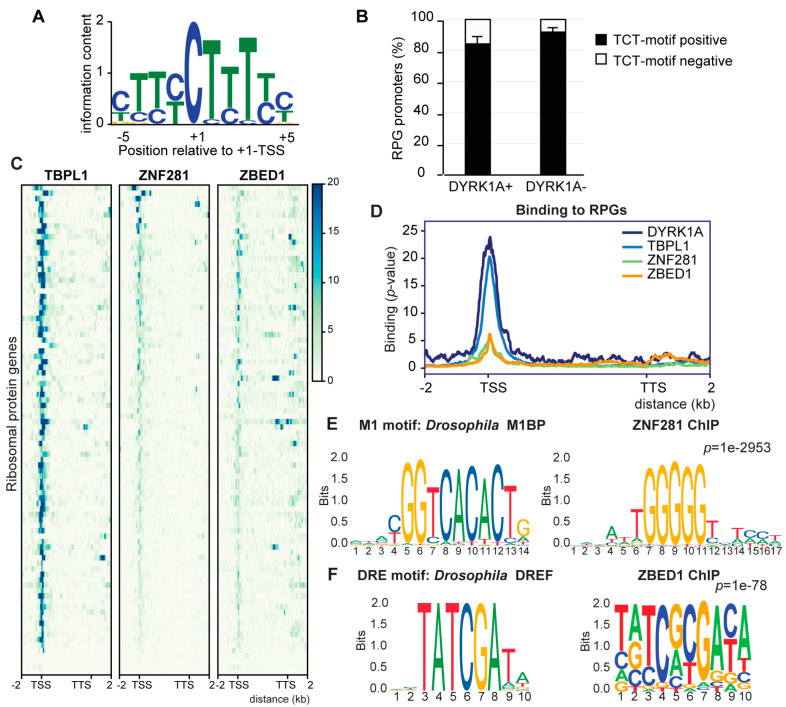
Analysis of the homologs of *Drosophila* RPG expression regulators in mammalian cells. (**A**) Sequence logo of the TCT motif found in human RPGs. (**B**) Percentage of TCT-positive or negative human RPG promoters distributed according to the presence of DYRK1A (average ± stdev of DYRK1A occupancies in T98G, HeLa and U2OS cells). (**C**) Occupancy of human RPGs by TBPL1 (K562, ENCSR783EPA), ZNF281 (HepG2, ENCSR403MJY) and ZBED1 (K562, ENCSR286PCG). The genomic region considered is shown on the *x*-axis. (**D**) Occupancy of the transcription factors shown in panel **C** together with DYRK1A. (**E**) Sequence of the *Drosophila* M1 motif at Jaspar and of the DNA motif enriched in ZNF281-bound regions in human cells. (**F**) Sequence of the DRE motif in *Drosophila* at Jaspar and of the motif enriched in all ZBED1-bound regions in human cells.

Next, we wondered whether the RPG promoters that bind DYRK1A were characterized by any other feature. Most studies on the regulation of RPG expression in higher eukaryotes have used *Drosophila* as a model system, identifying several sequence elements and TFs that regulate RPG transcription [13,14,15]. One of them is the TCT motif that is a specific promoter element for the expression of RPGs [13]. Using high-confidence human TSS data, we scanned the human RPG promoters and found the TCT consensus sequence (YC + 1TYTYY; Figure 3A) in 77 of the 86 promoters analyzed (Table S9). However, we did not find any correlation between the TCT motif and the presence of DYRK1A at these promoters (Figure 3B).

In *Drosophila*, the TCT element drives the recruitment of Pol II and, consequently, the transcription of RPGs through the coordinated action of TRF2, but not of TBP, and the TF motif 1 binding protein (M1BP) and DNA-replication-related element (DRE) factor (DREF) [14,15]. Thus, we asked whether the transcriptional regulators of the RPGs were functionally conserved between *Drosophila* and humans, and, if so, how they might be related to the DYRK1A promoter association. We analyzed the presence of the homologs of the fly TFs at the human RPG promoters: TBPL1, the homolog of TRF2; zinc finger protein 281 (ZNF281), the homolog of M1BP; and zinc finger BED-type containing 1 (ZBED1), the homolog of DREF. This analysis showed that TBPL1 binds to the promoter of most RPGs (68 out of 90 in K562 cells; Figure 3C,D; Table S9), similar to its behavior in *Drosophila* [14]; no particular enrichment was detected based on the presence or absence of a TATA box (Figure S3A). The presence of TBPL1 was detected at DYRK1A-positive and negative promoters (Figure S3B), and the results from an unbiased clustering analysis did not allow for the detection of the differential binding of TBPL1 at RPG promoters positive for DYRK1A (Figure S3C). ZNF281 was only detected at seven RPG promoters (Figure 3C; Table S9); in this regard, the sequence motif enriched within the ZNF281 ChIP-Seq dataset differed from the M1BP binding motif (http://jaspar.genereg.net/matrix/MA1459.1/ (accessed on 1 January 2021); Figure 3E) [10]; thus, we cannot assume that M1BP and ZNF281 are fully functional homologs. ZBED1 binds to several human RPG promoters and gene bodies (Figure 3C; Table S9), and the motif enriched in the ChIP-Seq dataset partially overlaps with the *Drosophila* DREF motif (http://jaspar.genereg.net/matrix/MA1456.1/ (accessed on 1 January 2021); Figure 3E). However, no enrichment for this motif was found within the human RPG promoters. ZBED1 might recognize the TCTCGCGAGA motif [40], yet the CMEA analysis did not find a unimodal and centered distribution of the TCTCGCGAGA motif within the ZBED1 ChIP peaks (Figure S3D). Hence, ZBED1 did not appear to bind directly to the TCTCGCGAGA motif, which is consistent with data suggesting that the TCTCGCGAGA motif and the human DRE are distinct regulatory elements [38]. No information on a general transcriptional effect of ZBED1 on RPGs is available other than that its depletion reduced the transcription of *RPS6*, *RPL10A* and *RPL12* genes in human foreskin fibroblasts [40]. Finally, ZBED1 binding to human RPGs showed no particular correlation with the presence of DYRK1A (Figure S3E). Together, these data suggest that there is little functional conservation of the core promoter elements of RPGs between *Drosophila* and humans, either in cis or trans. Furthermore, the association to chromatin of the TF homologs to those identified in *Drosophila* does not appear to depend on the presence of the TCTCGCGAGA motif or the binding of DYRK1A to human RPG promoters.

### 3.4. GABP and DYRK1A Are Differentially Distributed at the RPG Promoters

**Figure 4:**
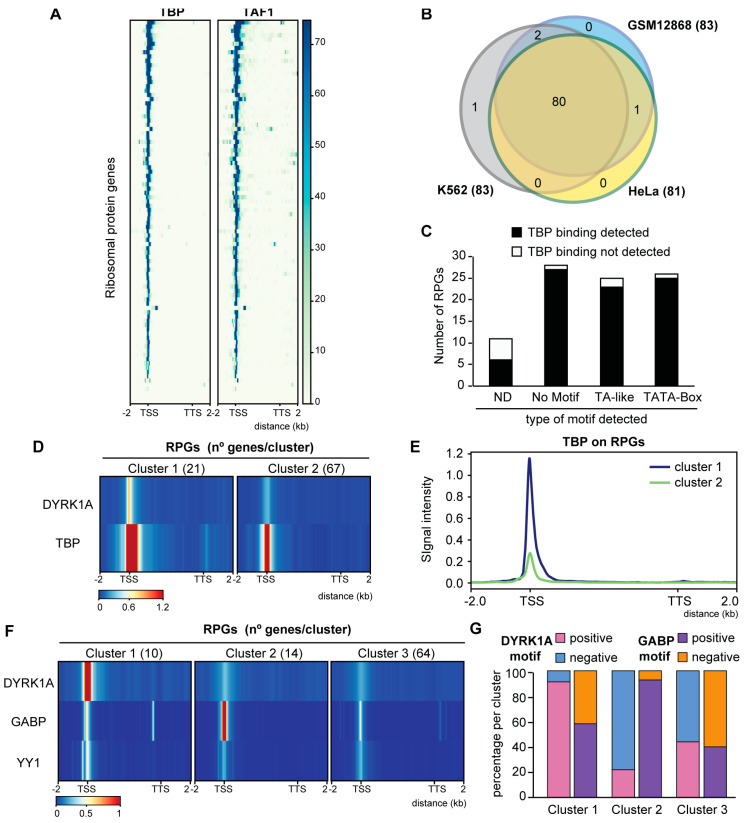
Analysis of TFs whose binding sites are over-represented at human RPG promoters. (**A**) TBP and TAF1 occupancy in human RPGs, showing the genomic region considered on the *x*-axis (K562 cells, GSE31477 and ENCSR000BKS). (**B**) Overlap of TBP occupancy at RPGs in different human cell lines (GSE31477). (**C**) Relationship of the presence of a TATA box or TA-like sequences in human RPG promoters according to Perry et al. (2005) [16] and TBP binding in HeLa cells (ND, not determined). (**D**) Unbiased *k*-mean clustering of the average binding of DYRK1A (HeLa, this work) and TBP (HeLa, GSE31477) on human RPGs. The color scale bar indicates the binding score and the genomic region considered is shown on the *x*-axis. (**E**) Metagene plot showing TBP occupancy relative to human RPGs in HeLa cells according to the clusters shown in Figure 4D. The *y*-axis represents the relative protein recruitment quantified as ChIP-Seq reads. (**F**) Unbiased *k*-mean clustering of the average binding of DYRK1A (T98G, this work), GABP and YY1 (SK-N-SH, GSE32465) to the RPGs. The color scale bar indicates the binding score and the genomic region considered is shown on the *x*-axis. (**G**) Percentage of RPG promoters containing a DYRK1A or a GABP motif in each of the clusters shown in Figure 4F.

We then asked whether DYRK1A recruitment was associated with the presence of TFs whose binding sites are known to be over-represented at human RPG promoters, such as TBP, MYC, SP1, GABP and YY1. Instead of using motif occurrence, as in previous studies analyzing RPG promoter architecture [11,16], we took advantage of genome-wide mapping data for each of the TFs. Thus, though TBP was predicted to be differentially enriched at the human RPG promoters based on the presence of the TATA box [16], the analysis of TBP occupancy found TBP bound to nearly all human RPG promoters (93%) in the different cell lines (Figure 4A,B; Table S9). Therefore, TBP appears to be a general component of the human RPG transcriptional machinery, irrespective of the presence of a TATA consensus, a TATA-like sequence or the complete absence of such motifs (Figure 4C). Consistent with a role for TBP in the assembly of the preinitiation complex (PIC) at the RPG promoters, the presence of the TFIID subunit TBP-associated factor 1 (TAF1) was strongly correlated with that of TBP (Figure 4A; Table S9). Notably, we observed stronger TBP binding at the DYRK1A-enriched RPG promoters than at the RPG promoters devoid of DYRK1A (Figure 4D,E and Figure S4A), suggesting that these two factors might cross-talk.

The presence of YY1, SP1 and MYC was also detected in almost all RPG promoters irrespective of the presence of cognate binding sites (Figure S4B; Table S9), and an unbiased clustering analysis showed no differential distribution of any of these factors based on the presence of DYRK1A (Figure S4C). By contrast, DYRK1A and GABP were distributed into one cluster that included those RPG promoters with a high DYRK1A occupancy and a low GABP presence (Figure 4F and Figure S4D, cluster 1), and another in which promoters were depleted of DYRK1A with a strong GABP occupation (Figure 4F and Figure S4D, cluster 2). Indeed, while the DYRK1A-associated TCTCGCGAGA motif was mostly over-represented in cluster 1, the GABP motif is a hallmark of cluster 2 (Figure 4G). These results suggest that the presence of DYRK1A labels a specific subset of RPGs that might respond distinctly to those labeled by GABP.

### 3.5. The Expression of RPGs Is Sensitive to DYRK1A Depletion

**Figure 5:**
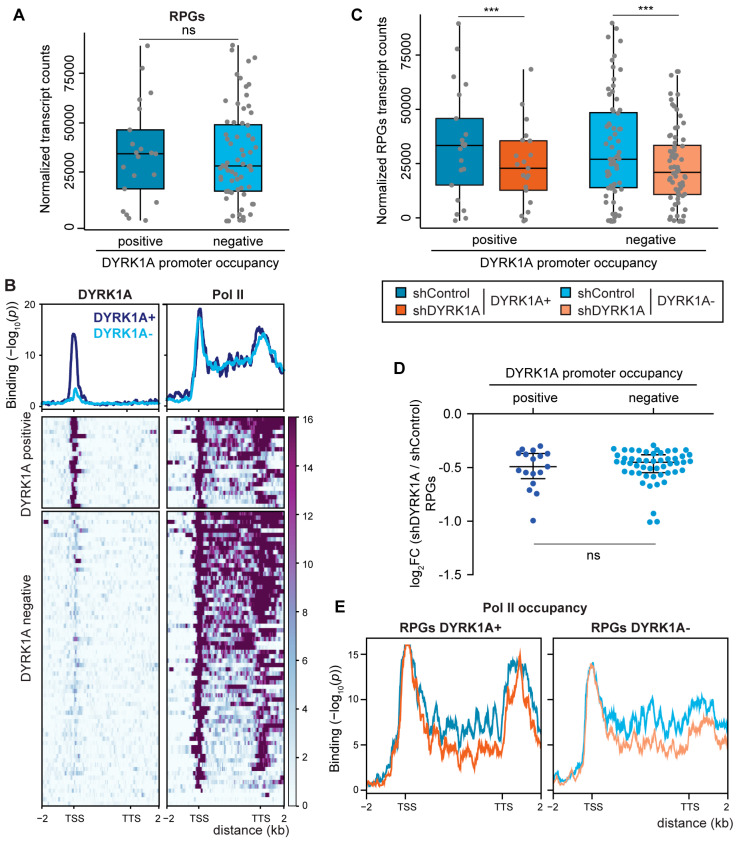
Depletion of DYRK1A causes a general reduction in RPG expression. (**A**) RNA levels of human RPGs (normalized counts) separated into two clusters according to the DYRK1A presence at their promoters (unpaired two-tailed Mann–Whitney test, ns = not significant). (**B**) Bottom panel, DYRK1A and Pol II chromatin occupancy of human RPGs depicted as metagenes and separated into two clusters according to the presence/absence of DYRK1A at the promoters. The binding score (−log_10_ Poisson *p*-value) is indicated by the color scale bar. Top panel, density plot corresponding to the mean value of the heatmap (blue and light-blue lines correspond to DYRK1A-positive or DYRK1A-negative RPGs, respectively). The binding score (−log_10_ Poisson *p*-value) is indicated on the *y*-axis. (**C**) Expression of RPGs (normalized counts) in T98G cells classified according to the presence (positive) or absence (negative) of DYRK1A at their promoters, and comparing shRNA Control cells (blue and light-blue, respectively) or shDYRK1A (orange and light-orange, respectively; Wilcoxon matched-pairs signed-ranks test, *** *p* < 10^−5^). The reduction in DYRK1A is shown in Figure S7B. (**D**) The graph presents the log_2_FC for the RPG mRNAs (adj *p*-value < 0.05) in shDYRK1A vs. shControl and clusterized according to DYRK1A presence at their promoters (two-tailed Mann–Whitney test, ns = not significant). (**E**) Density plots corresponding to the mean value of Pol II binding in RPGs clusterized as positive and negative for DYRK1A binding in shControl (blue and light-blue lines) and shDYRK1A conditions (orange and light-orange lines).

Based on the ability of DYRK1A to regulate transcription when recruited to chromatin [20], we wondered whether the transcription of the RPGs might be modulated by DYRK1A. Indeed, the TCTCGCGAGA motif is a cis element for the regulation of the expression of several RPGs, such as *RPL7A* [41], *RPS6*, *RPL10A*, *RPL12* [40] and *RPS11* [20]. Furthermore, we demonstrated that the *RPS11* promoter responds to DYRK1A in a kinase- and motif-dependent manner [20]. As described in *Drosophila* [14], the transcripts of most human RPGs were in the group of the top 5% most strongly transcribed genes in the cell lines analyzed (Figure S5A–F). Globally, the expression of genes that have promoters occupied by DYRK1A is stronger than that of the genes in which DYRK1A is absent (Figure S5G). The same tendency towards a stronger expression of DYRK1A-bound targets was observed when assessing the RPGs, although the differences were not statistically significant in any of the cell lines analyzed (Figure 5A and Figure S5H,I). The ChIP-Seq data of Pol II for RPGs revealed a profile corresponding to actively transcribed genes, with Pol II occupancy detected all along the gene bodies, with the exception of those RPGs not expressed in the cell line analyzed (Figure 5B). No differences in the distribution of Pol II were found between the DYRK1A-positive and negative RPGs (Figure 5B).

We assessed next whether the absence of DYRK1A affected the expression of its target genes by comparing the RNA expression of cells infected with a control lentivirus or a lentivirus expressing a shRNA to DYRK1A, and we used *Drosophila* RNA for the spike-in normalization. The majority of the differentially expressed genes were downregulated in response to DYRK1A depletion (Figure S6A). Indeed, these downregulated genes were enriched in the subset of genes with DYRK1A at their promoters (Figure S6B), supporting a role for DYRK1A in transcriptional activation. A general reduction in RPG transcripts was observed in DYRK1A-silenced cells (Figure 5C), and the analysis indicated that RPGs whose promoters were occupied by DYRK1A and those without DYRK1A were affected to a similar extent (Figure 5C,D), suggesting that the effect of DYRK1A on RPG expression goes beyond its direct binding to promoters. The reduction in the transcript levels of the selected DYRK1A target and nontarget RPGs was validated by RT-qPCR using two different shRNAs directed against DYRK1A (Figure S7A,C). The analysis of the occupancy of Pol II at the RPGs showed a general decrease in Pol II along the RPG gene bodies upon DYRK1A depletion at both the DYRK1A-positive and DYRK1A-negative RPGs (Figure 5E), pointing to a transcriptional effect as responsible for the reduction in the RPG transcript steady-state levels. All these results depict a complex scenario in which the physiological levels of DYRK1A are important for maintaining RPG mRNA levels through at least two different, although not necessarily exclusive, mechanisms: on the one hand, through its recruitment to the proximal promoter regions of a subset of RPGs; on the other hand, through a not-yet-identified mechanism that impacts the levels of transcribing Pol II at the RPGs.

### 3.6. DYRK1A Depletion Causes a Reduction in the Ribosome Content

**Figure 6:**
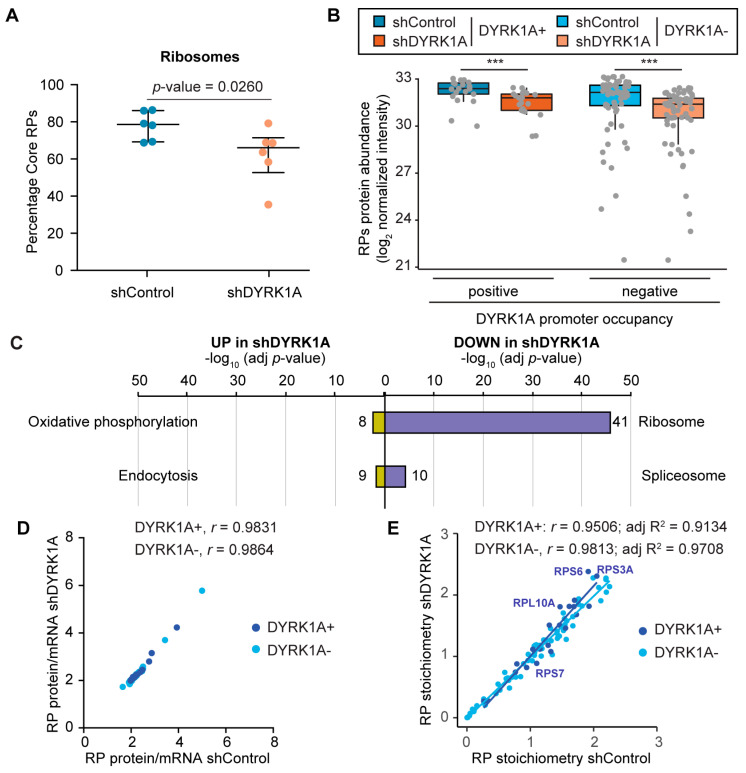
DYRK1A depletion causes a reduction in the ribosome content. (**A**) The relative RP values (the sum of all RP TOP3 values/the sum all TOP3 values) for each sample of shControl (blue) and shDYRK1A (brown) T98G cells are represented, also showing the median and IQRs (n = 6, two-tailed Mann–Whitney test). (**B**) Levels of the RPs (log_2_ of normalized peptide intensities) separated into two clusters according to the presence of DYRK1A at the promoters of their corresponding genes, both in shDYRK1A and shControl T98G cells (Wilcoxon matched-pairs signed-rank test, *** *p* < 10^−4^). The reduction in DYRK1A is shown in Figure S9E. (**C**) Functional categorization of the proteins found more abundant in the shDYRK1A cells (UP) or in the shControl cells (DOWN) (proteins with a *p*-value < 0.05 in the comparisons or present in only one of the conditions were used). The number of proteins identified in each category is shown; see also Table S10. (**D**,**E**) Correlation analysis of the ratio of RP protein and mRNA abundances (**D**) and RP stoichiometry (**E**) of the shControl and shDYRK1A T98G cells. RP stoichiometry is defined as the intensity of each RP relative to the intensity of all RPs. A color code was used to indicate the presence (+)/absence (−) of DYRK1A at the RPG promoter regions. The Spearman’s correlation coefficient is shown for each subset. In (**E**), the value for the adjusted R2 for each set is also included.

We next wondered whether the downregulation of the RPG transcript levels induced by DYRK1A silencing would be reflected at the protein level. As such, we used MS to quantify the ribosome composition in the control cells and in DYRK1A-depleted cells using a cytosolic fraction enriched in ribosomes from T98G cells (see Materials and Methods for details). Our dataset had a strong overlap with other studies defining the human riboproteome [42,43] (Figure S8A) with the biological functions enriched related to protein synthesis (Figure S8B; Table S10). Other functions, such as oxidative phosphorylation, RNA transport/processing or endocytosis, probably reflect cosedimenting complexes; the enrichment in nuclear proteins related with splicing has also been described [44]. Nevertheless, core RPs represent more than 75% of the protein mass in the fraction analyzed (Figure 6A).

The MS data allowed for the detection and quantification of all RPs except for RPL10L and RPL39L, which showed low mRNA levels or which were not expressed at all in T98G cells, and for RPL41 that is usually not detected by MS approaches [42]. In addition, the data indicated a lower relative abundance of the RPS27L, RPL3L, RPL7L1, RPL22L1, RPL26L1 and RPL36AL paralogs than their corresponding pairs (Figure S9A,B; Table S11), suggesting that they are under-represented in the ribosomes from T98G cells. However, we cannot rule out that the variation in stoichiometry could be due to some RPs being loosely bound to the ribosome; thus, their presence may be affected by the method for the cell extract preparation or because the pools of RPs performing extraribosomal functions might not be present in the cell fraction analyzed. We observed variability in the protein/mRNA ratio in the T98G cells, with extreme cases for most of the weakly expressed paralogs, like RPL3L, RPL7L1 or RPL22L1 (Figure S9C). Although we cannot rule out that some RPs are under-represented in the fraction analyzed, the results are consistent with published data showing that the amounts of RPs correlate poorly with their corresponding mRNA levels in other cellular contexts [45]. In agreement with the RNA data, our analysis did not reveal significant differences in the relative abundance of RPs encoded by DYRK1A-positive or DYRK1A-negative genes (Figure 6B).

We next focused on the alterations induced by silencing DYRK1A. The datasets revealed significant differences in proteins associated with specific functional categories in the ribosomal-enriched fractions from the control and DYRK1A-depleted cells. Proteins in the oxidative phosphorylation category were enriched in the shDYRK1A cell fraction (Figure 6C), including components of the mitochondrial electron transport chain (COX6B1, COX7A2L, SDHB) or mitochondrial ATP synthases (Table S10). Conversely, the ribosome category was enriched in the protein dataset reduced in the fraction from shDYRK1A cells (Figure 6C; Table S10). Indeed, DYRK1A depletion significantly reduced the total ribosome mass when calculated relative to the total amount of protein in the fraction analyzed by MS (Figure 6A). In accordance with the general effect of DYRK1A on the RPG transcript levels, less RP amounts were found for the RPGs containing or lacking DYRK1A at their promoters (Figure 6B and Figure 6B and Figure S9A,B). Notably, the rRNA content tended to fall (Figure S9D), supporting the decrease in the ribosome amounts. The protein/mRNA ratios correlated strongly between the control and DYRK1A-silenced cells (Figure 6D), suggesting that changes in the protein abundance were largely due to the changes in the RNA abundance upon DYRK1A depletion. Finally, no big differences in the RP stoichiometry were observed between the shControl and shDYRK1A cells (Figure 6E; RPL22L1, *p*-value = 0.022; RPL27, *p*-value = 0.0411; two-tailed unpaired Mann–Whitney). All these results indicate that cells respond to DYRK1A depletion by reducing the steady-state levels of the RPs, and that this effect occurs, at least in part, at the transcript level.

### 3.7. DYRK1A Plays a Role in Cell-Size Control by Regulating Protein Synthesis

**Figure 7:**
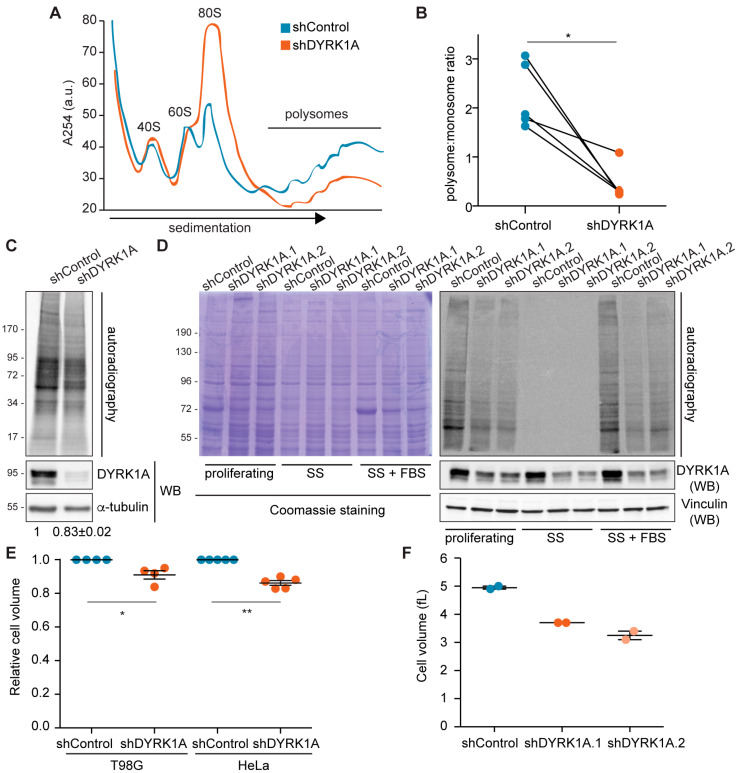
DYRK1A-dependent regulation of protein synthesis might impact cell size. (**A**) Polysome profile of T98G cells in shDYRK1A (orange line) and shControl (blue line) conditions. The position of the 40S, 60S, 80S and polysome peaks is indicated. The *y*-axis shows absorbance at 254 nm in arbitrary units and the *x*-axis corresponds to the different fractions. (**B**) The area under the curve for polysomes and monosomes was measured from the polysome profiles of paired shControl and shDYRK1A experiments, and the polysome:monosome ratio was calculated (the values for each condition in each biological replicate are represented with a color-coded dot and connected with lines; n = 5, shDYRK1A.1 [3] and shDYRK1A.2 [2]; Wilcoxon matched-pairs signed-ranks test, * *p* < 0.05). (**C**) Protein synthesis assays were performed by metabolic ^35^S-methionine labeling in shDYRK1A or shControl T98G cells. DYRK1A levels were analyzed by WB (Supplementary Materials). Quantification of the average radioactive intensity of independent experiments is shown at the bottom of the images (mean ± SEM, n = 3; Wilcoxon matched-pairs signed-ranks test, *p* < 10^−3^). (**D**) Autoradiography of protein extracts prepared from proliferating, serum-starved cells for 48 h (SS) or serum-starved cells reincubated with FBS for 30 min (SS + FBS) and pulse-labeled with ^35^S-Met for 20 min. Equal numbers of T98G cells infected with the indicated shRNA lentivectors were used. The reduction in DYRK1A was assessed in WB (Supplementary Materials) and a Coomassie-stained gel as a loading control is also shown. Cell cycle profiles are shown in Figure S10B. (**E**) Cell volume represented in arbitrary units with the control cells set as 1 (mean ± SEM; Mann–Whitney test, * *p* < 0.05, ** *p* < 0.01). (**F**) Cell volume of T98G cells infected with lentivirus expressing two independent shRNAs against DYRK1A or a shRNA Control (n = 2).

Altered ribosome biogenesis can lead to major defects in translation; thus, we assessed the impact of DYRK1A on the translational status of cells. The functional status of ribosomes was first analyzed by polysome profiling on sucrose gradients upon DYRK1A silencing. The downregulation of DYRK1A diminished the polysome fractions, which corresponded to those ribosomes engaged in active translation, with a concomitant increase in the monosome peak (Figure 7A,B). These results indicate that DYRK1A depletion leads to polysome disorganization. Indeed, there was a significant reduction in the translational rates in the DYRK1A-depleted cells when measured through radiolabeled ^35^S-methionine incorporation (Figure 7C). This effect was specific, as it was clearly observed with two distinct shRNAs targeted to DYRK1A (Figure 7D).

In eukaryotes, cell growth is coupled to cell cycle progression; therefore, the global translation rates change during the cell cycle [46]. Defects in the cell cycle have been associated with alterations in DYRK1A levels in different cellular environments [47]. Indeed, we found that DYRK1A silencing alters the cell cycle balance in T98G cells, augmenting the population of cells in the G1 phase (Figure S10A). Thus, we next checked whether the shift in the cell cycle phases was associated with the reduced translation rates. As such, the T98G cells were arrested in G1 by serum deprivation (Figure S10B), which led to a strong reduction in the rate of translation (Figure 7D). Serum addition for 30 min induced protein synthesis (Figure 7D), with no changes in the cell cycle profiles (Figure S10B). In these conditions, lower rates of translation persisted in the cells with silenced DYRK1A relative to the control cells (Figure 7D), suggesting that the reduction in protein synthesis upon DYRK1A silencing is independent of the alterations in the cell cycle.

As DYRK1A is a highly pleiotropic kinase, we wondered whether the reduction in translation could be due to an effect of DYRK1A on other signaling pathways that regulate protein synthesis. Therefore, we analyzed the effect of DYRK1A on one of the major signaling pathways that regulates protein translation, the mechanistic target of rapamycin (mTOR) pathway [48], assessing Thr389 phosphorylation of the RPS6 kinase beta-1 (RPS6KB1 or p70S6K) that represents a late event in mTOR pathway activation. DYRK1A depletion did not alter Thr389–p70S6K phosphorylation (Figure S10C,D); likewise, the MS data showed no differences in the amount of RPS6 peptides phosphorylated at Ser235, Ser236 and Ser240 (Figure S10E), all targets of p70S6K downstream of mTOR [48]. Accordingly, DYRK1A does not appear to affect the mTOR pathway under regular growth conditions. Other signaling pathways, like the cellular stress and unfolded protein response, can inhibit protein synthesis through the Ser51 phosphorylation of the translation initiation factor eIF2α [49]. However, the levels of Ser51–eIF2α phosphorylation remained unchanged in the absence of the DYRK1A (Figure S10F,G), indicating that the DYRK1A-dependent inhibition of translation is not mediated by eIF2α phosphorylation. Moreover, DYRK1A was not detected in the ribosome-enriched fraction by MS or in immunoblots of polysome-associated fractions (Figure S10H), suggesting that it is not tightly bound to actively translating ribosomes and that it probably does not act directly on polysomes. We would like to point out that our results are based on the behavior of tumor cell lines as experimental systems. Therefore, the functional interaction of DYRK1A with cell growth regulators in other physiological backgrounds cannot be excluded. In fact, a reduced soma size of cortical layer V neurons has been observed in a conditional *Dyrk1a* null mouse model, which was related to mTOR dysregulation [50].

Finally, as reduced protein synthesis might affect cell mass, we checked whether the cell size was affected by the loss of DYRK1A activity. Indeed, DYRK1A-silenced HeLa and T98G cells were both significantly smaller than their controls in terms of cell volume (Figure 7E,F). Hence, the fine-tuning of cellular DYRK1A levels is important to assure the proper size of human cells is maintained.

## 4. Discussion

In this study, we show that there is a subset of RPGs in mammals whose promoters are marked by the palindromic TCTCGCGAGA motif and the presence of the protein kinase DYRK1A, as shown by the chromatin recruitment analysis in different human and mouse cell lines. A motif enrichment analysis did not find the TCTCGCGAGA motif within the RPG promoters in yeast, basal metazoa or plants [11], although a similar motif (CGCGGCGAGACC) was found within the proximal promoter regions of 28 RPGs in *Caenorhabditis elegans* [12]. In *Drosophila*, no similar motifs were enriched in the RPG promoters [10], though the DRE sequence has been proposed to mirror such a motif [40]. By contrast, the TCTCGCGAGA motif is conserved in vertebrates and it is generally found in DNAse I-accessible regions [38,51], leading to the proposal that it serves as a core promoter element in TATA-less promoters associated with CpG islands [38]. These findings would suggest that the TCTCGCGAGA motif is a cis-regulatory element that arose later in evolution and that it might be linked to coevolution with regulatory factors acting in trans. Besides DYRK1A, the transcriptional repressor ZBTB33, the T-cell factors Tcf7l2 and Tcf1 and the BTG3-associated nuclear protein (BANP) have been shown to use the TCTCGCGAGA motif as a chromatin recruitment platform [39,51,52,53], and we confirm here the presence of ZBTB33 at the RPG promoters that contain this motif. Whether the TCTCGCGAGA motif interacting proteins compete or collaborate in the regulation of common target genes, including RPGs, is an issue that merits further exploration.

In *Drosophila*, RPG expression is regulated by a combination of two TFs, M1BP and DREF, which are associated with distinct subsets of RPGs through binding to their corresponding DNA-sequence motifs. These two proteins are responsible for recruiting TRF2, which substitutes for TBP in the assembly of the PIC [13,14,15]. Our analysis shows no evidence of such a regulatory network in humans. On the one hand, we observe TBP binding to almost all RPG promoters, regardless of whether or not they contain a TATA sequence, which indicates that TBP would be responsible for PIC assembly in human RPGs, as supported by the presence of the largest subunit of TFIID, TAF1. It is worth noting that the presence of DYRK1A at RPG promoters is correlated with more TBP binding, opening the question on the existence of a functional cross-talk between DYRK1A and TBP. TBPL1, a TRF2 homolog, also binds to almost all RPGs, consistent with the finding that TBPL1 is recruited to the PIC, not as a substitute for TBP but rather along with it, in mouse testis [54]. In *Drosophila*, the depletion of TBP or TBPL1 individually does not affect the expression of the TCT-promoter-bearing RPGs [55], suggesting that these two core TFs might function redundantly. Whether this is the case in mammalian cells is yet unknown. No correlation between DYRK1A presence at RPG promoters and more TBPL1 binding has been detected, though, in this case, we were unable to use datasets from the same cell type and we cannot exclude the impact of cell-type specificity. By contrast, we do not detect significant enrichment of the human M1BP and DREF homologs ZNF281 and ZBED1 at the human RPG promoters. Therefore, a completely different set of regulators must exist in mammals to interpret and to respond dynamically to growth and stress cues.

With the exception of the TCT motif, most of the sequences implicated in regulating the expression of RPGs in humans are only present in a subset of RPGs [13], including the binding motifs for the SP1, GABP, MYC and YY1 TFs. We have extended these results by analyzing the presence of these TFs at RPG promoter regions, revealing a more general distribution than that inferred through the presence of their binding sites. Therefore, no specific bias was found for the recruitment of TFs, like SP1, YY1 or MYC, to promoters containing the TCTCGCGAGA motif or positive for DYRK1A recruitment. By contrast, the RPGs that associate with DYRK1A are characterized by reduced GABP binding. The distinct distribution of DYRK1A and GABP could allow for the differential regulation of subsets of RPGs in response to a variety of stimuli. Notably, both *DYRK1A* and *GABP* are located on human chromosome 21 and, when in trisomy, their overexpression might contribute to the general increase in RPG mRNA transcripts in the brain of individuals with DS (Table S12). We are aware that a limitation of our study is that the comparative analysis of DYRK1A and TFs’ occupancies has been performed using data from different sources. When possible, datasets from the same cell line have been used (for instance, HeLa for TBP) or from a similar origin (for instance, neural cell lines T98G and SK-N-SH for GABP and YY1).

The depletion of the DYRK1A results in the downregulation of RPG transcripts, affecting both RPGs with DYRK1A bound at their promoters and RPGs devoid of DYRK1A. These results resemble those found with the manipulation of MYC levels, resulting in changes in RPG transcripts not directly associated to the presence of MYC at the promoter regions of those genes [56,57]. In the case of RPGs bound by DYRK1A within their promoter, this reduction could be a direct effect of the loss of DYRK1A at their proximal promoters and the subsequent reduction in Pol II CTD phosphorylation, as shown for *RPS11* [20], with the reduction in transcript levels being a combination of alterations at both the initiation and elongation steps. However, additional mechanisms must exist to explain that the decrease in the RPG mRNA levels occurs in a general manner. The reduction in Pol II occupancy at the RPGs upon DYRK1A depletion would suggest that transcription might be a target. Even so, we cannot exclude that the reduction in DYRK1A levels may induce post-transcriptional effects acting on RPG mRNA steady-state levels, or may alter the complex interactions between RPG mRNA synthesis/degradation and ribosome biogenesis that have been described in yeast directed to ensure fidelity in ribosome assembly [58,59,60]. Finally, the DYRK1A-dependent effect on increasing the population of cells in G1 could also be at play.

Our results demonstrate that the production of RPs and, ultimately, the number of ribosomes in proliferating cells depends on the physiological levels of DYRK1A. This could be a direct consequence of the alterations in the RPG transcript levels, since the protein/mRNA ratios are not significantly affected upon DYRK1A silencing. Additionally, compensating mechanisms at the level of ribosome biogenesis might also operate [1]. In either case, the shortage of ribosomes upon a loss of DYRK1A provokes translational dysfunction, with the cell size reduction as one of the possible phenotypic outputs. As mentioned above for the DYRK1A-dependent impact on the mRNA steady-state levels, the increase in the G1 population could also be a contributing factor, or, alternatively, a consequence of delayed growth; however, a DYRK1A-dependent reduction in cell size in postmitotic neurons has been described [50]. The reduction in ribosomes could either globally affect translation or may result in transcript-specific translational control. In this context, the pool of mRNAs associated with polysomes that respond differentially to DYRK1A downregulation still needs to be characterized. Together with the identification of cis-regulatory elements in these transcripts, this information will surely help to discriminate between the two possibilities and establish a mechanistic framework. Nonetheless, our findings do not rule out the existence of other DYRK1A-dependent effectors that contribute to altered ribosome biogenesis and/or translation: such a multilayer regulatory effect is not uncommon in growth regulators, as it is the case of the mTOR protein kinase [48].

The physiological context for the activity of DYRK1A on translational control remains a matter of speculation at this stage. For instance, DYRK1A has been associated with the regulation of cell proliferation, and given that cell growth and proliferation are intimately linked [46,61], this kinase could couple the cell cycle with protein synthesis by maintaining the amounts of RPs. In addition, DYRK1A plays essential roles in central nervous system development, not only influencing cell numbers but also their differentiation [21]. Indeed, a reduction in the size of the soma of cortical layer V neurons has been shown in conditional *Dyrk1a*-null mice [50]. The authors showed reduced mTOR-dependent signaling, but the RP accumulation dysregulation, as we have observed in tumor cell lines, might also represent a contributing factor. It is also possible that the effects of DYRK1A on cell mass may affect other tissues, particularly since heterozygous mouse models exhibit a global reduction in body size [62]. In this regard, DYRK1A has been shown to phosphorylate Pol II at gene loci involved in myogenic differentiation [30], a process that requires increased protein synthetic rates [46]. In a different context, defects in protein production are closely related to cancer, since enhanced translation is required to boost cell proliferation [3,4] and DYRK1A has both positive and negative effects on cell proliferation, depending on the tumor context [22,23]. Finally, mutations in specific RPGs produce very unique phenotypes [3], including craniofacial anomalies and urogenital malformations [63]. In addition, *RPL10* mutations have been linked to neurodevelopmental conditions, including autism spectrum disorders and microcephaly [64], and, indeed, translation is a process targeted in autism-associated disorders [65]. All of these features are hallmarks of *DYRK1A* haploinsufficiency syndrome in humans or in animal models with *Dyrk1a* dysregulation [26,66].

## 5. Conclusions

In summary, our findings have uncovered the protein kinase DYRK1A as a novel player in the regulation of translation and cell growth in mammals. This discovery adds complexity to our understanding of DYRK1A’s role in cellular biology. The study also raises questions about the mechanisms by which mammalian cells regulate the transcription of ribosomal protein genes, prompting comparisons with existing knowledge from studies in yeast or flies. Furthermore, the findings create opportunities for future investigations to explore the connections between the mechanistic aspects of DYRK1A activity and the pathological consequences that arise from its dysregulation. 

## Data Availability

All the raw and processed sequencing data generated in this study have been submitted to the NCBI GEO repository under accession number GSE155809. The raw proteomics data have been submitted to the Proteomics Identifications Database (PRIDE) under accession number PXD022966.

## Supplementary Materials

The following supporting information can be downloaded at: https://www.mdpi.com/article/10.3390/biom14010031/s1, Supplementary Material and Methods; Figure S1: Supporting data for Figure 1; Figure S2: The transcription factor ZBTB33 is recruited to RPG-positive promoters for DYRK1A and the TCTCGCGAGA DNA motif; Figure S3: Recruitment of TBPL1 and ZBED1 to human RPG promoters; Figure S4: Recruitment of TBP, MYC, SP1 and YY1 to RPG promoters in comparison with that of DYRK1A; Figure S5: RPG mRNA expression in different human cell lines; Figure S6: Global alterations in gene expression upon DYRK1A depletion; Figure S7: Supporting data for the alterations in RPG transcript levels in DYRK1A-depleted cells; Figure S8: Riboproteome analysis; Figure S9: RP expression analysis based on MS quantitative data in T98G comparing shControl and shDYRK1A. Figure S10: Supporting data for Figure 7. Table S1: Antibodies used in this research; Table S2: ENCODE datasets used in the analysis of chromatin occupancy; Table S3: Primers for ChIP-qPCR; Table S4: Primers for RT-qPCR; Table S5: DYRK1A binding to RPGs in *Homo sapiens*; Table S6: DYRK1A binding to RPGs in *Mus musculus;* Table S7: RPGs within bidirectional transcription pairs; Table S8: ZBTB33 binding to RPGs in *Homo sapiens;* Table S9: TF binding to RPGs in *Homo sapiens;* Table S10: KEGG data from Figure 6C and Figure S8B; Table S11: RP MS data; Table S12: Differential RPG expression in Down syndrome individuals. References [13,20,33,35,37,67,68,69,70,71,72,73,74,75,76,77,78,79,80] are cited in Supplementary Materials.
