# Supplementary material for: Loss of the DYRK1A Protein Kinase Results in the Reduction in Ribosomal Protein Gene Expression, Ribosome Mass and Reduced Translation
Source: Biomolecules. 2023 Dec 25;14(1):31. doi: 10.3390/biom14010031 (PMC10813206; doi:10.3390/biom14010031)
Supplement: Supplementary file 1 [file biomolecules-14-00031-s001.zip › 1_Supplementary_Information.pdf]

Loss of the DYRK1A protein kinase results in reduction of ribosomal protein genes expression, ribosome mass and reduced translation

Chiara Di Vona*, Laura Barba, Roberto Ferrari and Susana de la Luna*

*, To whom correspondence should be addressed: Chiara Di Vona, chiara.divona@crg.eu. Susana de la Luna, susana.luna@crg.eu.

SUPPLEMENTARY INFORMATION

Supplementary Material and Methods

Figure S1: Supporting data for Figure 1.

Figure S2: The transcription factor ZBTB33 is recruited to RPG promoters positive for DYRK1A and the TCTCGCGAGA DNA-motif.

Figure S3: Recruitment of TBPL1 and ZBED1 to human RPG promoters.

Figure S4: Recruitment of TBP, MYC, SP1 and YY1 to RPG promoters in comparison with that of DYRK1A.

Figure S5: RPG mRNA expression in different human cell lines.

Figure S6: Global alterations in gene expression upon DYRK1A depletion.

Figure S7: Supporting data for the alterations in RPG transcript levels in DYRK1A depleted cells.

Figure S8: Riboproteome analysis.

Figure S9: RP expression analysis based on MS quantitative data in T98G comparing shControl and shDYRK1A.

Figure S10: Supporting data for Figure 7.

Tables S1-S4: Supplementary Tables for Material and Methods.

Table S5: DYRK1A binding to RPGs in *Homo sapiens.*

Table S6: DYRK1A binding to RPGs in *Mus musculus.*

Table S7: RPGs within bidirectional transcription pairs.

Table S8: ZBTB33 binding to RPGs in *Homo sapiens.*

Table S9: TF binding to RPGs in *Homo sapiens.*

Table S10: KEGG data from Figure 6C and Figure S8B.

Table S11: RP mass spectrometry data.

Table S12: Differential RPG expression in Down syndrome individuals.

SUPPLEMENTARY MATERIALS AND METHODS

Cell culture and lentivirus-mediated transduction

The HEK-293T, HeLa, U2OS and T98G cell lines were obtained from the American Type Culture Collection (www.atcc.org). E14 (129/Ola) mouse embryonic stem cells (mESCs) were kindly provided by Maria Pia Cosma's laboratory (CRG). Human cells were cultured in Dulbecco's Modified Eagle's Medium (DMEM) supplemented with 10% (v/v) fetal bovine serum (FBS) and antibiotics (100 U/mL penicillin and 100 μg/mL streptomycin). mESCs were cultured in 0.1% gelatin coated plates, with DMEM supplemented with 15% FBS, 2 mM L–glutamine, 1 mM sodium pyruvate, 0.1 mM non-essential amino acids, 0.5 mM 2-mercaptoethanol, 1000 U/mL ESGRO recombinant mouse Leukemia Inhibitory Factor and antibiotics. For serum starvation, T98G cells were grown in DMEM without FBS for 16-48 h. All cell lines were grown at 37 ºC and in a 5% CO_2 atmosphere.

Lentiviral stocks were generated by transfecting HEK-293T cells by the calcium phosphate precipitation method with pCMV-VSV-G (Addgene #8454) envelope plasmid, pCMV-dR8.91 packaging construct, and pLKO-based Sigma Mission plasmids expressing short hairpin (sh)RNAs, either a non-targeting vector (shControl, Sigma #SHC016) or two shRNAs directed against DYRKA: DYRK1A.1 (TRCN0000022999) and DYRK1A.2 (TRCN0000199464). Lentivirus-containing supernatants were harvested 48 h and 72 h after transfection, and concentrated by ultracentrifugation (87,500 *xg*, 2 h, 4 ºC). For cell transduction, the virus was added to the medium with the cells in suspension, and replaced with DMEM 24 h after infection and seeding. Infected cells were selected by adding 1.25 μg/ml of puromycin (Sigma) for 48-72 h, and the cells were allowed to recover in the absence of puromycin for 24 h.

Cell cycle profile and cell volume determination

T98G cell pellets were fixed by dropwise addition of cold 70% ethanol and their DNA was stained with 4',6-diamino-2-phenylindole (1 μg/mL, Roche), 0.1% Triton X-100 in phosphate-buffered saline (PBS). Cells were analyzed with a LSR II flow cytometer (Becton Dickinson) with FACSDiva™ software v6.1.2 (Becton Dickinson). Cell volume was determined with the Beckman Coulter Z2 Cell and Particle Counter. Cells were harvested by trypsinization, washed in cold PBS and measured in triplicate. Cell-type dependent cursor settings were used to discriminate between vital and dead cells/cell debris.

Western blotting

To prepare the total cell lysates, cells were resuspended in SDS-lysis buffer (25 mM Tris-HCl pH 7.5, 1% sodium dodecyl sulfate [SDS], 1 mM ethylenediaminetetraacetic acid [EDTA], 10 mM $Na_4P_2O_7$, 20 mM β-glycerol phosphate) and heated for 10 min at 98 ºC. The protein extracted was quantified with the BCA Protein Assay Kit (Pierce-Thermo Scientific). For Western blotting (WB), cell lysates were resolved on SDS-polyacrylamide gels (SDS-PAGE) and transferred onto Hybond-ECL nitrocellulose membranes (Amersham Biosciences). The membranes were blocked for 1 h at room temperature with 10% non-fat milk (Cell Signaling Technologies) diluted in TBS-T (10 mM Tris-HCl pH 7.5, 100 mM NaCl, 0.1% Tween-20) and then incubated for 16 h at 4 ºC with the primary antibodies (Table S1) diluted in TBS-T containing 5% non-fat milk (or bovine serum albumin [BSA] for the phosphospecific antibodies). After several washes in TBS-T, the membranes were incubated with horseradish peroxidase-conjugated secondary antibodies for 1 h at room temperature diluted in TBS-T containing 5% non-fat milk. After washing in TBS-T, the chemiluminiscence signal was revealed with Western Lightning® Plus ECL (Perkin Elmer) and measured in a LAS-3000 image analyzer (Fuji PhotoFilm) with the LAS3000-Pro software. Band intensities were quantified with the ImageQuant™ TL software (GE Healthcare Life Sciences) or ImageStudio™ Lite (Li-Cor), and the relative protein levels were calculated using α-tubulin or vinculin as loading controls.

Mass spectrometry (MS)

Sample preparation: Ribosome-enriched pellets were washed three times with 100 mM ammonium bicarbonate (ABC) and resuspended in 6 M Urea-100 mM ABC. Extracts (10 μg) were incubated for 1 h at 37 ºC in the presence of 0.3 mM DTT, followed by incubation with 0.6 mM iodoacetamide (Sigma) for 30 min at room temperature in the dark. Proteins were digested for 16 h at 37 ºC with Lys-C endoprotease (1 μg: Wako) and then incubated for 8 h at 37 ºC with sequence-grade trypsin (1 μg: Promega). Peptides were desalted on Ultra Micro Spin Columns C18 (The Nest Group INC) prior to liquid chromatography (LC)-MS/MS.

Chromatographic and MS analysis: Samples were analyzed on a LTQ-Orbitrap Fusion Lumos mass spectrometer (Thermo Scientific) coupled to an Easy-nLC 1000 (Thermo Scientific, Proxeon) at the CRG/UPF Proteomics Unit. Peptides were loaded onto the analytical column and separated by reversed-phase chromatography on a 50 cm column with an inner diameter of 75 μm, packed with 2 μm C18 particles (Thermo Scientific). Chromatographic gradients started at 95% buffer A (0.1% formic acid in water) and 5% buffer B (0.1% formic acid in acetonitrile) with a flow rate of 300 nL/min for 5 min, and they gradually increased to 78% buffer A - 22% buffer B in 79 min, and then to 65% buffer A - 35% buffer B in 11 min. After each analysis, the column was washed with 10% buffer A - 90% buffer B for 10 min. The mass spectrometer was operated in positive ionization mode with nanospray voltage set at 2.4 kV and the source temperature at 275 °C. Ultramark 1621 was used for external calibration of the FT mass analyzer and an internal calibration was performed using the background polysiloxane ion signal at m/z 445.1200. Acquisition was performed in data dependent acquisition (DDA) mode and full MS scans with 1 micro scans were used at a resolution of 120,000 over a mass range of m/z 350-1500. Auto gain control (AGC) was set to 1E5 and charge state filtering that disqualifies single charged peptides was activated. In each cycle of DDA analysis, the most intense ions above a threshold ion count of 10,000 were selected for fragmentation following each survey scan. The number of selected precursor ions for fragmentation was determined by the "Top Speed" acquisition algorithm with a dynamic exclusion of 60 s. Fragment ion spectra were produced via high-energy collision dissociation at a normalized collision energy of 28% and they were acquired in the ion trap mass analyzer. AGC was set to 1E4, using an isolation window of 1.6 m/z and a maximum injection time of 200 ms. All data were acquired with Xcalibur software v4.1.31.9. Digested BSA (New England Biolabs) was analyzed between each sample to avoid sample carry over and to assure the stability of the instrument. QCloud [67] was used to control the instrument's longitudinal performance. The spectra acquired were analyzed with the Proteome Discoverer software suite (v2.3, Thermo Fisher Scientific) and the Mascot search engine (v2.6, Matrix Science) [68]. The data was used to search the SwissProt human database (February 2020), including a list of common contaminants and the corresponding decoy entries.

ChIP-Seq

Sample preparation: Formaldehyde-crosslinked cells were washed twice with cold PBS, resuspended in 1 mL of Lysis buffer I (5 mM PIPES pH 8.0, 85 mM KCl, 0.5% NP-40, protease inhibitor cocktail [cOmplete Mini, Roche Diagnostic]) and incubated on ice for 10 min. After centrifugation (800 *xg*, 5 min, 4 ºC), the cell pellets were resuspended in 0.6 mL of Lysis buffer II (1% SDS, 10 mM EDTA, 50 mM Tris-HCl pH 8.0, protease inhibitor cocktail)

and incubated for additional 10 min on ice. Chromatin was sonicated to an average size of 0.2-0.5 kb with a Bioruptor (Diagenode) and the chromatin corresponding to 150 μg DNA was diluted in 1 mL of Buffer III (300 mM NaCl, 200 mM Tris-HCl pH 8, 10 mM EDTA, 1% SDS, 5% Triton X-100) and the samples were incubated with specific antibodies (Table S1) or control rabbit IgGs (Santa Cruz sc-2027) for 16 h at 4 °C with rotation. The immunocomplexes were recovered by incubating with 30 μl of Protein A-Sepharose beads (GE Healthcare) for 3 h at 4 °C with rotation, and the beads were then washed three times with low salt buffer (50 mM HEPES pH 7.4, 140 mM NaCl, 1% Triton X-100), once with high salt buffer (50 mM HEPES pH 7.4, 500 mM NaCl, 1% Triton X-100), once with LiCl buffer (10 mM Tris-HCl pH 8, 250 mM LiCl, 1% NP-40, 1% deoxycholic acid, 1 mM EDTA), and once with TE buffer (100 mM Tris-HCl pH 7.5, 50 mM EDTA). All wash buffers were supplemented with the protease inhibitor cocktail. DNA was eluted by incubation in elution buffer (1% SDS, 100 mM $NaHCO_3$) in two steps of 30 min, each at 65 ºC, and crosslinking was reverted by an additional incubation in 200 mM NaCl for 16 h at 65 ºC. Finally, chromatin-associated proteins were degraded by adding Proteinase K (1.6 U, New England Biolabs) for 2 h at 45 ºC, and the DNA was purified by phenol/chloroform extraction and ethanol-precipitation. The DNA was quantified with the Qubit® dsDNA HS Assay Kit (Thermo Fisher Scientific).

Library generation and sequencing: DNA libraries were generated with the Ovation® Ultralow Library System V2 (NuGEN) and sequenced on an Illumina Hiseq-2500 sequencer at the CRG Genomics Unit, with 50 bp single end reads. The quality of the sequenced reads was controlled using with FastQC (www.bioinformatics.babraham.ac.uk/projects/fastqc). Reads were mapped to the human or mouse genome assemblies (hg38 and mm9, respectively) using Bowtie [69], and *p*-values for the significance of ChIP-Seq counts were calculated relative to the input DNA [33], using a threshold of 10^{-8} and FDR <1%.

RNA-Seq

Sample preparation: RNA was isolated with the RNeasy extraction kit and samples were treated for 30 min at 37 ºC with DNase I (2 U/μL, Ambion). RNA was quantified with NanoDrop and quality-controlled on a Bioanalyzer 2100 (Agilent). For T98G cells spike-in normalization, an equal number of T98G cells for each condition were mixed with a fixed number of *Drosophila melanogaster* Kc167 cells (1:4 ratio). For reverse transcription RT, 0.5-1 μg RNA was subjected to cDNA synthesis with Superscript II Reverse Transcriptase (Invitrogen) and random primers.

***Library generation and sequencing**:* Libraries were prepared with the TruSeq Stranded mRNA Sample Prep Kit v2 and sequenced with Illumina Hiseq-2500 to obtain 125 bp pair-ended reads.

***Data analysis**:* Genomic reads were mapped and counted using STAR [70], plotting T98G reads against the human and fly genomes (hg38 and dm3, respectively) or HeLa reads against the human genome alone. Those reads that could not be uniquely mapped to just one region were discarded. Differential gene expression was assessed with the DESeq2 package in R, filtering genes that had >10 average normalized counts per million [35]. In the case of T98G RNA-Seq, the size factor of each replicate was calculated according to exogenous *Drosophila* spike-in reads. Expression was considered to be altered when adj *p*-value ≤0.05, and a $\log_2$FC above 0.7 and below -0.7 for up- and downregulated genes, respectively. To measure RPG expression levels in U2OS cells, the GSE117212 dataset was analyzed in the same manner (GSM3274834, GSM3274835, GSM3274837; [71]), obtained from the NCBI's Gene Expression Omnibus (GEO) repository (https://www.ncbi.nlm.nih.gov/geo/). To analyze the expression of RPGs in Down syndrome individuals, the GEO dataset GSE5390 [72] was analyzed with GEO2R (https://www.ncbi.nlm.nih.gov/geo/geo2r/) using the default parameters.

Other computational tools

The Integrated Genomics Browser v9.0.0 [73] was used to visualize the ChIP-Seq data sets and an in-house gene and promoter annotation pipeline was used for peak annotation. The script uses data from a consensus position weight matrix (PWM) for the presence of the DYRK1A motif [20], and the University of California Santa Cruz (UCSC) genome browser software (http://genome.ucsc.edu/) via the UCSC Table Browser for Ensembl/NCBI gene and transcript annotation. Extra annotations were provided using Biomart [74]. BEDTools [75] was used to create overlaps of significant peaks with genomic annotated regions with the "BEDtools intersect" command. Density plots and heatmaps to profile the average binding of the factors analyzed on defined genomic intervals were generated with computeMatrix and plotHeatmap tools from deepTools v3.0.0 [76]. Four genes (*RPL10L*, *RPL3L*, *RPS18* and *RPS4Y2*) were excluded from the analysis because they were not bound by any of the factors. The DYRK1A-PWM [20] was used to define RPG promoters containing the DYRK1A consensus motif within a -1000 bp to +100 bp region from TSS using the FIMO package [77] with *p*-values ≤ $3x10^{-4}$. The *p*-value was converted to a *q*-value following the method of Benjamini and Hochberg [78]. *Bona-fide* motifs were considered when *p*-value < 10^{-4} and degenerated motifs were those with 10^{-4} < *p*-value < $3X10^{-4}$. CentriMo [37] was used to identify the significant preference of the DYRK1A motif for particular locations within DYRK1A binding RPGs over a ±250 bp region of ChIP-Seq peaks

summit. To identify RPG promoters harboring the TCT-motif [13], sequences from -50 to +50 (relative to the C+1 transcription start site) were retrieved from the Eukaryotic Promoter Database EPD (https://epd.epfl.ch//index.php; accessed on 1 January 2021) and analyzed using the MEME tool in the MEME Suite (http://meme-suite.org). The Jaspar database [79] was used to retrieve known transcription factor binding profiles (http://jaspar.genereg.net/; accessed on 1 January 2021). The Jaspar-associated Myc-PWM MA0147.3 was used to define RPG promoters containing the Myc motif within a -1000 bp to +100 bp region from TSS using the FIMO package [77] with *p*-values ≤ $1x10^{-4}$. The Enrichr webtool (https://maayanlab.cloud/Enrichr/; [80]) was used to identify pathways enriched in the proteomics datasets (KEGG 2019 Human). BioVenn (https://www.biovenn.nl/) was used to overlap gene/protein targets from different datasets. Human orthologs of the *Drosophila* proteins were found in FlyBase (release FB2018_06; https://flybase.org/).

Scatter and box plots were generated with the R package ggplot2. For the box plots, the bottom and top of the box are the first and third quartiles, and the band inside the box is the median. The ends of the whiskers represent the lowest data point still within a 1.5 interquartile range (IQR) of the lower quartile, and the highest data point still within 1.5 IQR of the upper quartile. Any data not included between the whiskers is plotted as an outlier with a dot. Bar graphs were generated with Microsoft Excel v15.33.

SUPPLEMENTARY REFERENCES

13. Parry, T.J.; Theisen, J.W.; Hsu, J.Y.; Wang, Y.L.; Corcoran, D.L.; Eustice, M.; Ohler, U.; Kadonaga, J.T. The TCT motif, a key component of an RNA polymerase II transcription system for the translational machinery. *Genes Dev* **2010**, *24*, 2013-2018, doi:10.1101/gad.1951110.
20. Di Vona, C.; Bezdan, D.; Islam, A.B.; Salichs, E.; Lopez-Bigas, N.; Ossowski, S.; de la Luna, S. Chromatin-wide profiling of DYRK1A reveals a role as a gene-specific RNA polymerase II CTD kinase. *Mol Cell* **2015**, *57*, 506-520, doi:10.1016/j.molcel.2014.12.026.
33. Ferrari, R.; Su, T.; Li, B.; Bonora, G.; Oberai, A.; Chan, Y.; Sasidharan, R.; Berk, A.J.; Pellegrini, M.; Kurdistani, S.K. Reorganization of the host epigenome by a viral oncogene. *Genome Res* **2012**, *22*, 1212-1221, doi:10.1101/gr.132308.111.
35. Love, M.I.; Huber, W.; Anders, S. Moderated estimation of fold change and dispersion for RNA-seq data with DESeq2. *Genome Biol* **2014**, *15*, 550, doi:10.1186/s13059-014-0550-8.
37. Bailey, T.L.; Machanick, P. Inferring direct DNA binding from ChIP-seq. *Nucleic Acids Res* **2012**, *40*, e128, doi:10.1093/nar/gks433.

67. Chiva, C.; Olivella, R.; Borras, E.; Espadas, G.; Pastor, O.; Sole, A.; Sabido, E. QCloud: A cloud-based quality control system for mass spectrometry-based proteomics laboratories. *PLoS One* **2018**, *13*, e0189209, doi:10.1371/journal.pone.0189209.
68. Perkins, D.N.; Pappin, D.J.; Creasy, D.M.; Cottrell, J.S. Probability-based protein identification by searching sequence databases using mass spectrometry data. *Electrophoresis* **1999**, *20*, 3551-3567.
69. Langmead, B.; Trapnell, C.; Pop, M.; Salzberg, S.L. Ultrafast and memory-efficient alignment of short DNA sequences to the human genome. *Genome Biol* **2009**, *10*, R25, doi:10.1186/gb-2009-10-3-r25.
70. Dobin, A.; Davis, C.A.; Schlesinger, F.; Drenkow, J.; Zaleski, C.; Jha, S.; Batut, P.; Chaisson, M.; Gingeras, T.R. STAR: ultrafast universal RNA-seq aligner. *Bioinformatics* **2013**, *29*, 15-21, doi:10.1093/bioinformatics/bts635.
71. Bluhm, A.; Viceconte, N.; Li, F.; Rane, G.; Ritz, S.; Wang, S.; Levin, M.; Shi, Y.; Kappei, D.; Butter, F. ZBTB10 binds the telomeric variant repeat TTGGGG and interacts with TRF2. *Nucleic Acids Res* **2019**, *47*, 1896-1907, doi:10.1093/nar/gky1289.
72. Lockstone, H.E.; Harris, L.W.; Swatton, J.E.; Wayland, M.T.; Holland, A.J.; Bahn, S. Gene expression profiling in the adult Down syndrome brain. *Genomics* **2007**, *90*, 647-660, doi:10.1016/j.ygeno.2007.08.005.
73. Nicol, J.W.; Helt, G.A.; Blanchard, S.G., Jr.; Raja, A.; Loraine, A.E. The Integrated Genome Browser: free software for distribution and exploration of genome-scale datasets. *Bioinformatics* **2009**, *25*, 2730-2731, doi:10.1093/bioinformatics/btp472.
74. Durinck, S.; Moreau, Y.; Kasprzyk, A.; Davis, S.; De Moor, B.; Brazma, A.; Huber, W. BioMart and Bioconductor: a powerful link between biological databases and microarray data analysis. *Bioinformatics* **2005**, *21*, 3439-3440, doi:10.1093/bioinformatics/bti525.
75. Quinlan, A.R.; Hall, I.M. BEDTools: a flexible suite of utilities for comparing genomic features. *Bioinformatics* **2010**, *26*, 841-842, doi:10.1093/bioinformatics/btq033.
76. Ramirez, F.; Ryan, D.P.; Gruning, B.; Bhardwaj, V.; Kilpert, F.; Richter, A.S.; Heyne, S.; Dundar, F.; Manke, T. deepTools2: a next generation web server for deep-sequencing data analysis. *Nucleic Acids Res* **2016**, *44*, W160-165, doi:10.1093/nar/gkw257.
77. Grant, C.E.; Bailey, T.L.; Noble, W.S. FIMO: scanning for occurrences of a given motif. *Bioinformatics* **2011**, *27*, 1017-1018, doi:10.1093/bioinformatics/btr064.
78. Benjamini, Y.; Hochberg, Y. Controlling the False Discovery Rate: a practical and powerful approach to multiple testing. *Journal of the Royal Statistical Society. Series B* **1995**, *57*, 289-300.
79. Fornes, O.; Castro-Mondragon, J.A.; Khan, A.; van der Lee, R.; Zhang, X.; Richmond, P.A.; Modi, B.P.; Correard, S.; Gheorghe, M.; Baranašić, D., et al. JASPAR 2020:

update of the open-access database of transcription factor binding profiles. *Nucleic Acids Res* **2020**, *48*, D87-92, doi:10.1093/nar/gkz1001.

80. Kuleshov, M.V.; Jones, M.R.; Rouillard, A.D.; Fernandez, N.F.; Duan, Q.; Wang, Z.; Koplev, S.; Jenkins, S.L.; Jagodnik, K.M.; Lachmann, A., et al. Enrichr: a comprehensive gene set enrichment analysis web server 2016 update. *Nucleic Acids Res* **2016**, *44*, W90-97, doi:10.1093/nar/gkw377.
