# Supplementary material for: Loss of the DYRK1A Protein Kinase Results in the Reduction in Ribosomal Protein Gene Expression, Ribosome Mass and Reduced Translation
Source: Biomolecules. 2023 Dec 25;14(1):31. doi: 10.3390/biom14010031 (PMC10813206; doi:10.3390/biom14010031)
Supplement: Supplementary file 1 [file biomolecules-14-00031-s001.zip › 2_Supplementary Figures.pdf]

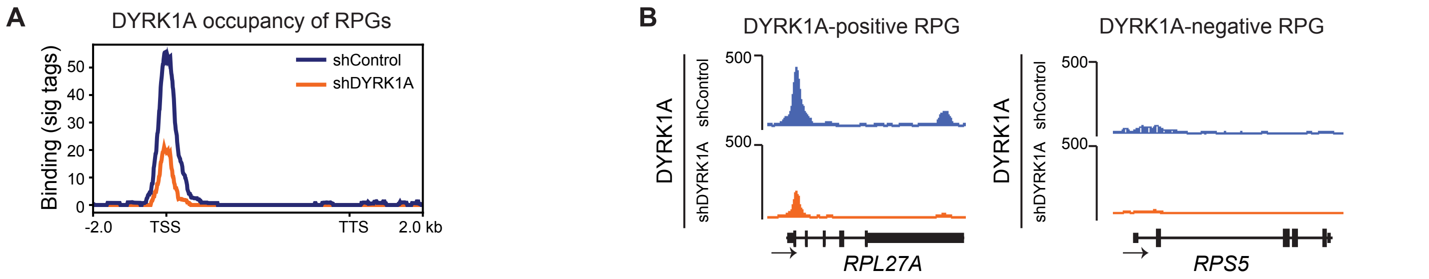

Figure S1. Supporting data for Figure 1.
(**A**) Metagene plot showing DYRK1A occupancy of human RPGs in shControl (blue) or shDYRK1A (orange) infected T98G cells. The *y*-axis represents the relative protein recruitment, quantified as significant (sig) ChIP-Seq tags.
(**B**) Representative example of DYRK1A occupancy at a DYRK1A binding (*RPL27A*) or a DYRK1A-negative (*RPS5*) RPG in T98G cells infected with the shControl or shDYRK1A lentivirus. The arrow indicates the direction of transcription.

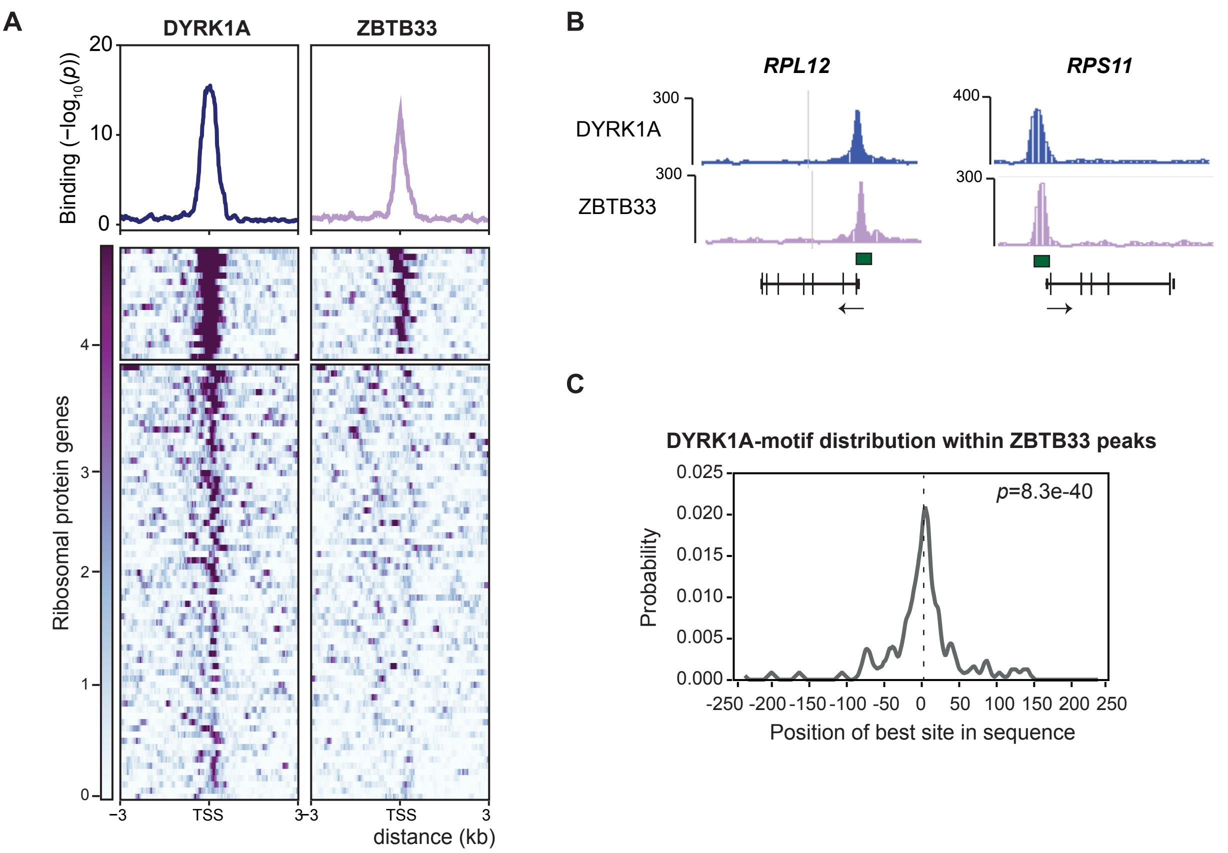

Figure S2. The transcription factor ZBTB33 is recruited to RPG promoters positive for DYRK1A and the TCTCGCGAGA DNA-motif.
(**A**) Density plots showing DYRK1A and ZBTB33 ChIP binding relative to the TSS of all human RPGs. The heatmaps show DYRK1A and ZBTB33 occupancy across all human RPGs relative to the TSS in T98G cells. The binding score ($-\log_{10}$ Poisson *p*-value) is indicated by the color scale bar and the offset was ± 3 kb from the TSS. (**B**) Representative examples of RPGs occupied by DYRK1A and ZBTB33. The arrows indicate the direction of transcription. (**C**) CentriMo plot showing the distribution of the DYRK1A motif in the RPG promoters that bind ZBTB33. The solid curve shows the positional distribution of the best strong DYRK1A motif site at each position in the ZBTB33 RPG-ChIP-Seq peaks (500 bp). The *p*-value for the central motif enrichment is indicated.

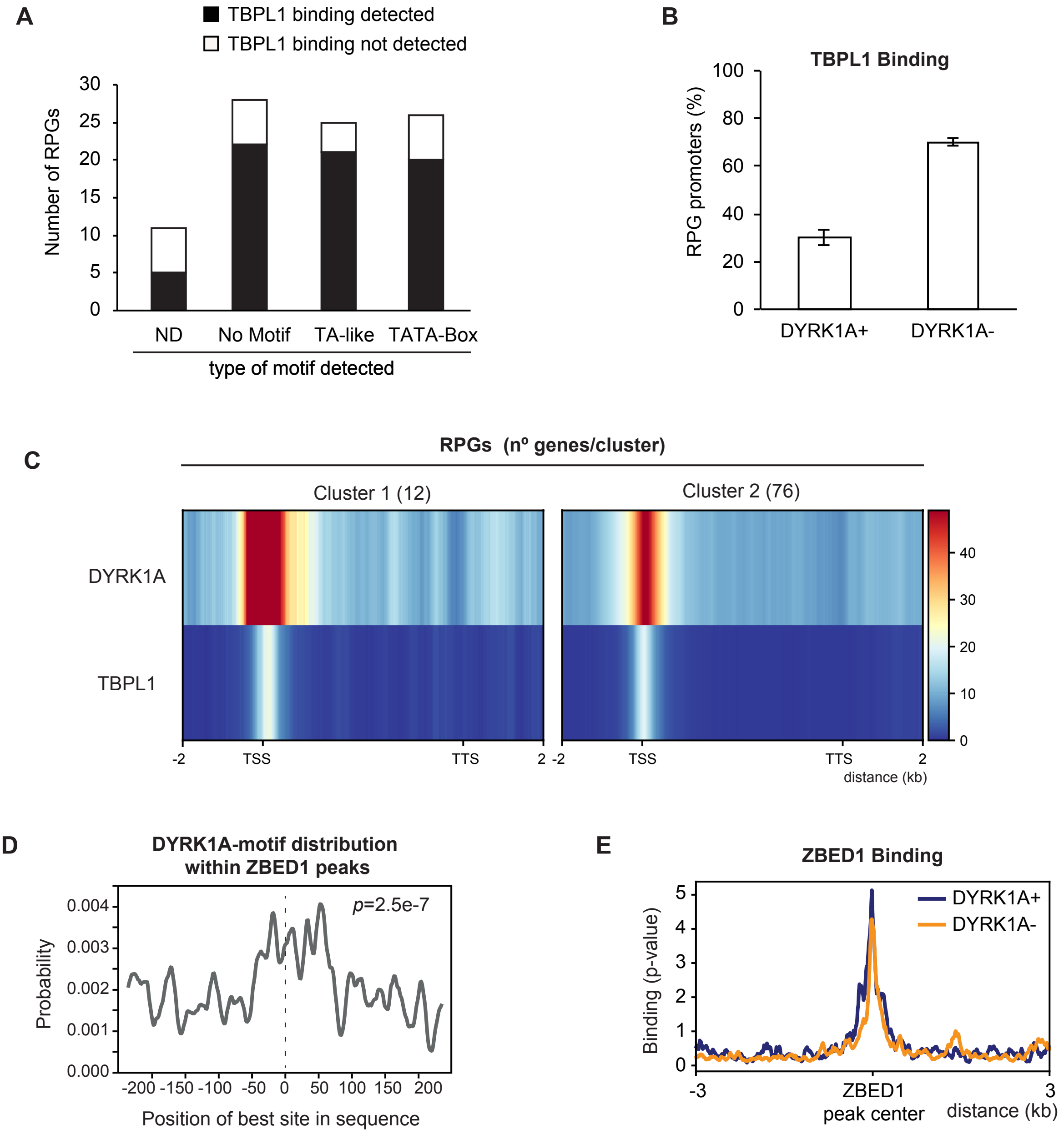

Figure S3. Recruitment of TBPL1 and ZBED1 to human RPG promoters.

(**A**) Relationship of the presence of a TATA-box or TA-like sequences in human RPG promoters according to Perry et al., (2005) and TBPL1 binding in K562 cells (ND, not determined). (**B**) Percentage of human RPG genes with the presence of TBPL1 at their promoters (K562, ENCSR783EPA) distributed according to the presence of DYRK1A (average ± stdev of DYRK1A occupancies in T98G, HeLa and U2OS cells). (**C**) Unbiased *k*-mean clustering of the average binding of DYRK1A (T98G, this work) and TBPL1 (K562, ENCSR783EPA) on human RPGs. The color scale bar indicates the binding score and the genomic region considered is shown on the *x*-axis. (**D**) CentriMo plot showing the distribution of the DYRK1A motif for the RPG promoters that bind ZBED1 (K562, ENCSR286PCG). The solid curve shows the positional distribution (averaged over bins 10 bp in width) of the best strong site of the DYRK1A motif at each position in the RPG-ChIP-Seq peaks (500 bp). The *p*-value for the central motif enrichment is indicated. (**E**) Density plot of the ZBED1 occupancy at bound RPGs (42 out of 90 RPGs) relative to center of the ZBED1 peaks. ZBED1-bound RPGs were classified as either DYRK1A positive or negative.

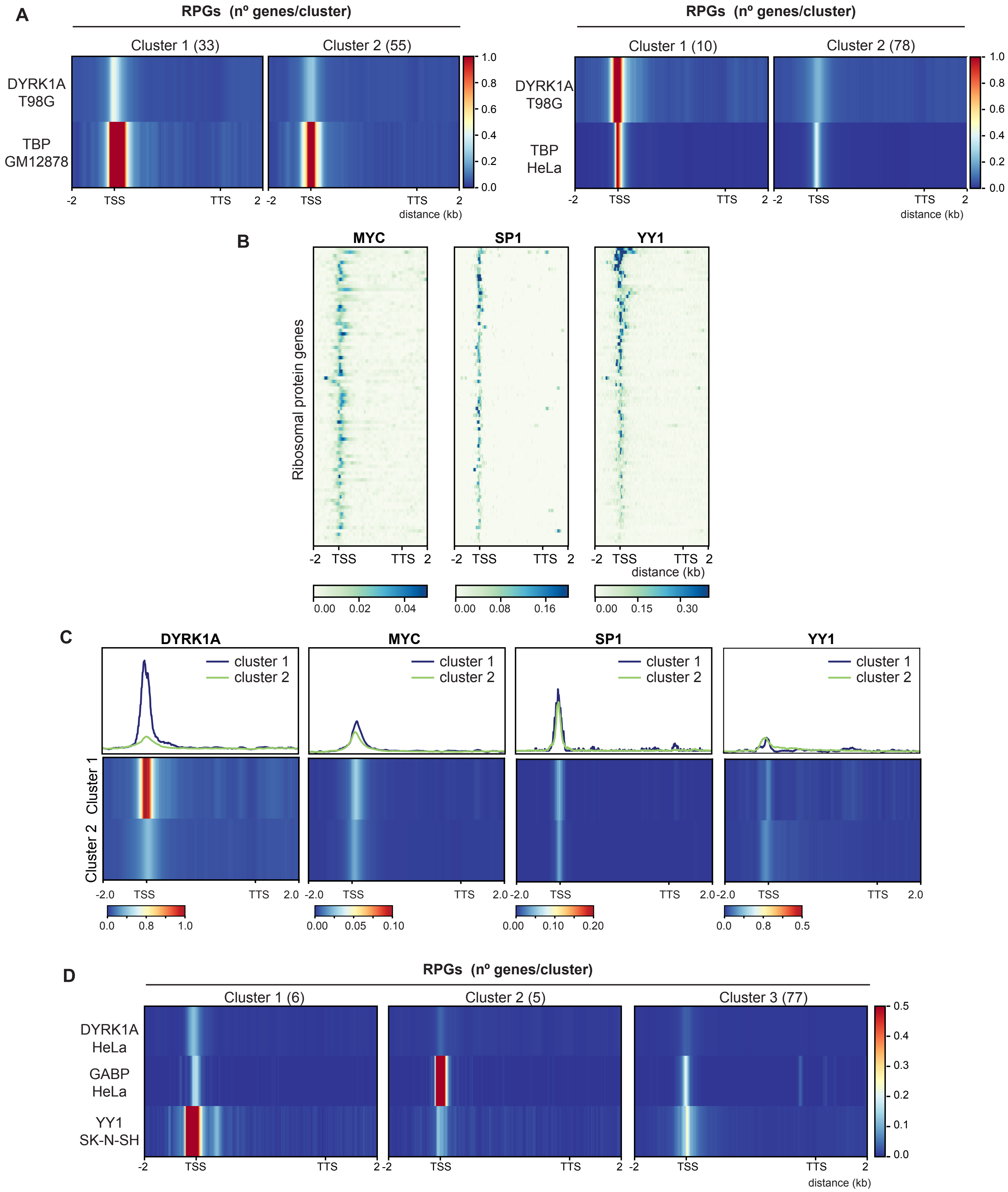

Figure S4. Recruitment of TBP, MYC, SP1 and YY1 to RPG promoters in comparison with that of DYRK1A.
(**A**) Unbiased *k*-mean clustering of the average binding of DYRK1A (T98G, this work) and TBP (GM12878 or HeLa, as indicated: GSE31477) on human RPGs. The color scale bar indicates the binding score and the genomic region considered is shown on the *x*-axis. (**B**) The heatmaps show MYC, SP1 and YY1 (GM12868, GSE33213 and GSE32465) occupancy on human RPGs. The genomic region considered is shown on the *x*-axis and the color scale bar indicates the binding score. (**C**) Unbiased *k*-mean clustering of the average binding of DYRK1A (T98G cells, this work), MYC, SP1 and YY1 (GM12868, GSE33213 and GSE32465) to RPGs. The color scale bar indicates binding score. (**D**) Unbiased k-mean clustering of the average binding of DYRK1A (HeLa, this work), GABP (HeLa, GSE32465) and YY1 (SK-N-SH, GSE32465) to RPGs. The color scale bar indicates the binding score and the genomic region considered is shown on the x-axis.

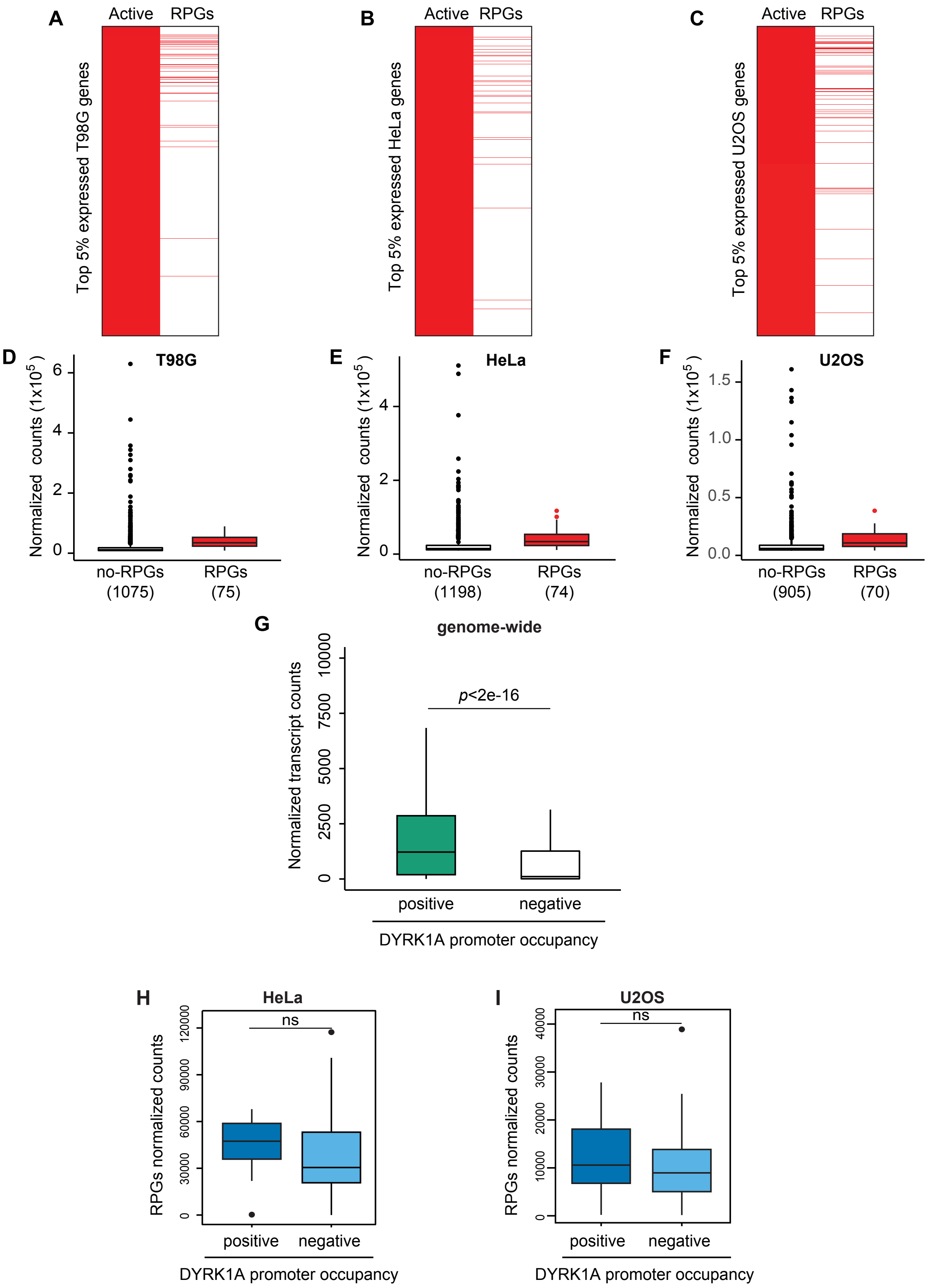

Figure S5. RPG mRNA expression in different human cell lines. (**A-C**) The heatmaps correspond to the top 5% more expressed genes in T98G (**A**), HeLa (**B**) and U2OS (**C**). RPGs are highlighted on the right-hand side in each panel (normalized read counts from RNA-Seq experiments: T98G and HeLa, this work; U2OS, GSE117212). (**D-F**) Box plots for the expression of genes corresponding to the 5% of the most strongly expressed genes, with RPGs removed (no-RPGs) or only considering RPGs (RPGs) in T98G (**D**), HeLa (**E**) and U2OS (**F**). (**G**) RNA levels of all human genes expressed in T98G cells (normalized counts) separated into two clusters depending on the presence of DYRK1A at their promoters (unpaired two-tailed Student's test). (**H, I**) Box plot indicating RPG transcript levels (normalized counts) separated into two clusters depending on the presence of DYRK1A at their promoters based on RNA-Seq data from HeLa (**G**, this work) or U2OS (**H**, GSE117212) (unpaired Mann-Whitney test, ns=not significant).

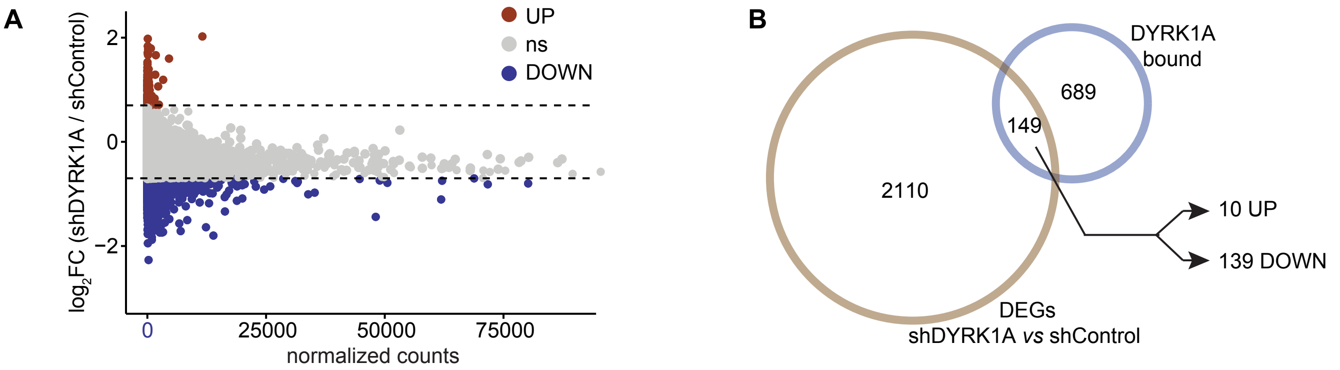

Figure S6. Global alterations in gene expression upon DYRK1A depletion. (**A**) Differentially expressed genes in T98G upon DYRK1A silencing as analyzed by RNA-Seq after spike-in normalization (adj *p*-value < 0.05; DOWN, $\log_2(FC) < -0.7$; UP, $\log_2(FC) > 0.7$; ns, not significant). Dotted lines indicate the thresholds of statistical significance. (**B**) Overlap between differentially expressed genes (DEG) as determined by RNA-Seq and genes with DYRK1A at their promoters witnessed by ChIP-Seq in T98G cells.

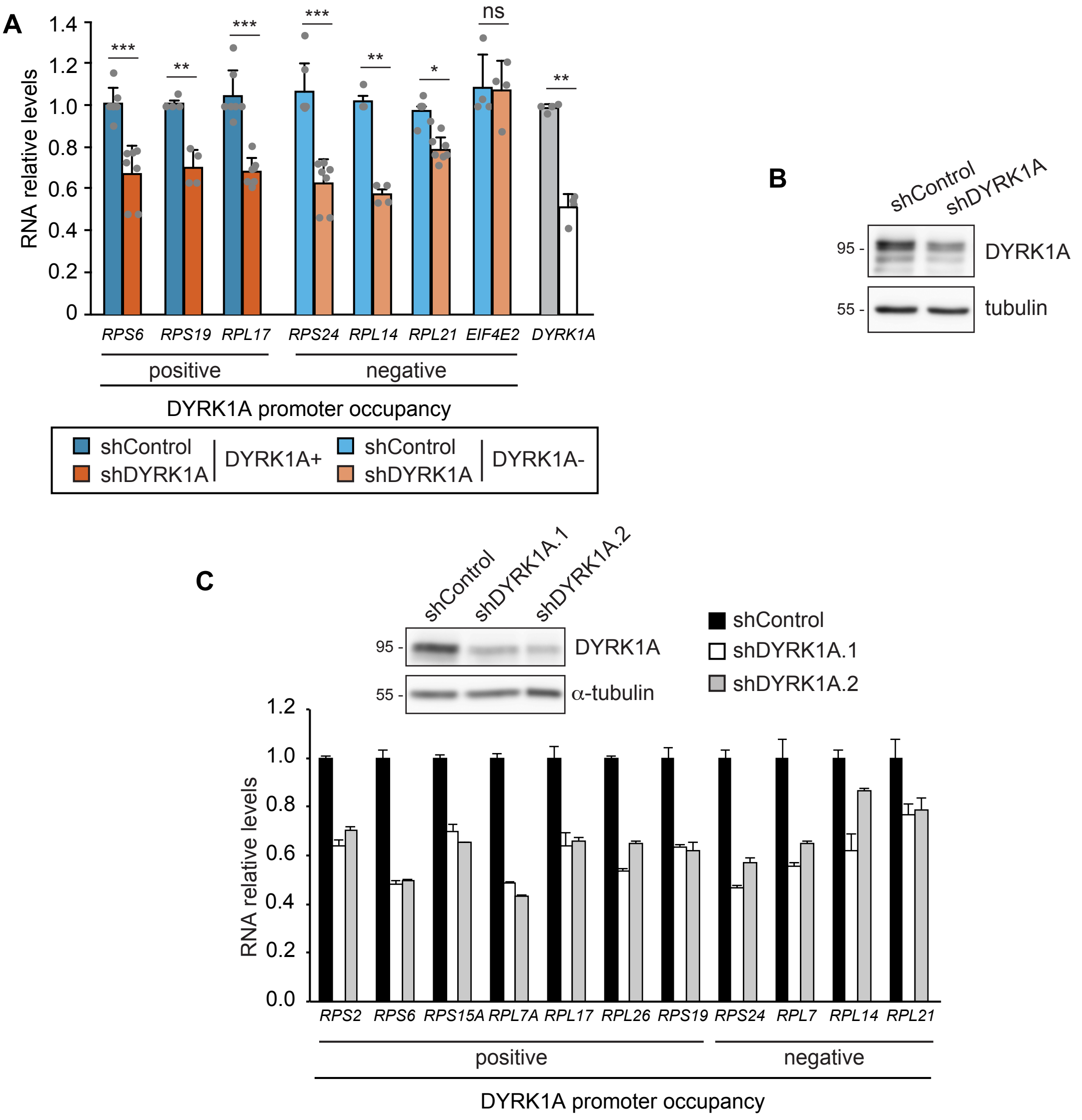

Figure S7. Supporting data for the alterations in RPG transcript levels in DYRK1A depleted cells. (**A**) RT-qPCR of RPGs in T98G transduced with shControl or shDYRK1A lentiviruses. *DYRK1A* and a translation-related non-RPG factor (*EIF4E2*) RNA levels are also shown. The data were corrected by *Drosophila* spike-in values (mean ± SD of independent experiments; Mann-Whitney test, *$p \leq 0.05$, **$p \leq 0.01$, ***$p \leq 0.001$, ns=not significant). (**B**) Levels of DYRK1A in WBs of samples shown in Figure 5C. (**C**) RT-qPCR of selected RPGs in T98G upon DYRK1A downregulation with two distinct shRNAs (mean ± SD of 3 technical replicates).

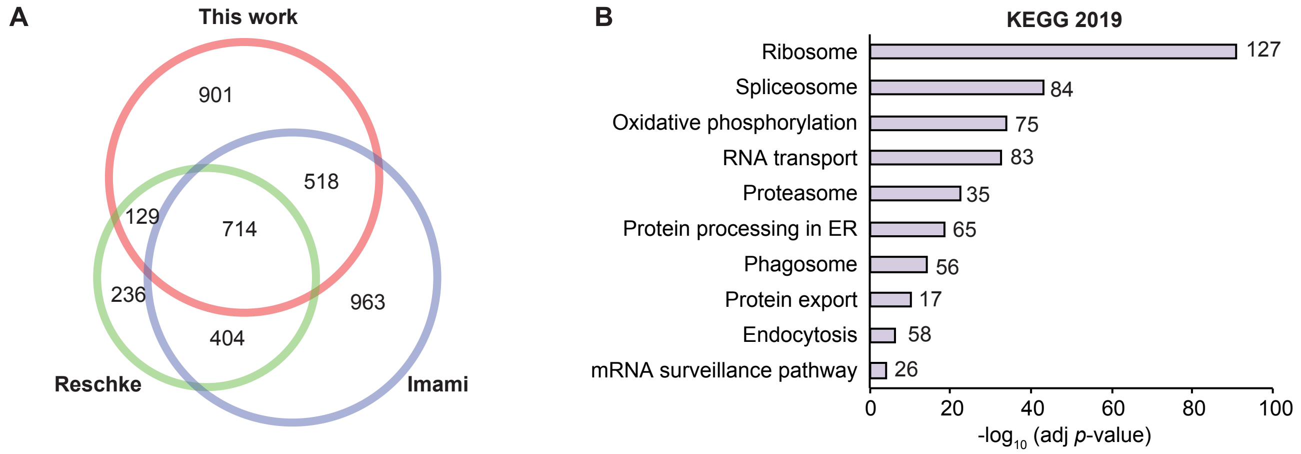

Figure S8. Riboproteome analysis. (**A**) Venn diagram indicating the overlap between the riboproteomes identified in Reschke et al (2013) and Imami et al (2018), and the proteins identified by MS in this work. (**B**) Functional categorization of the proteins identified by MS, showing the number of proteins identified in each category (see also Table S10).

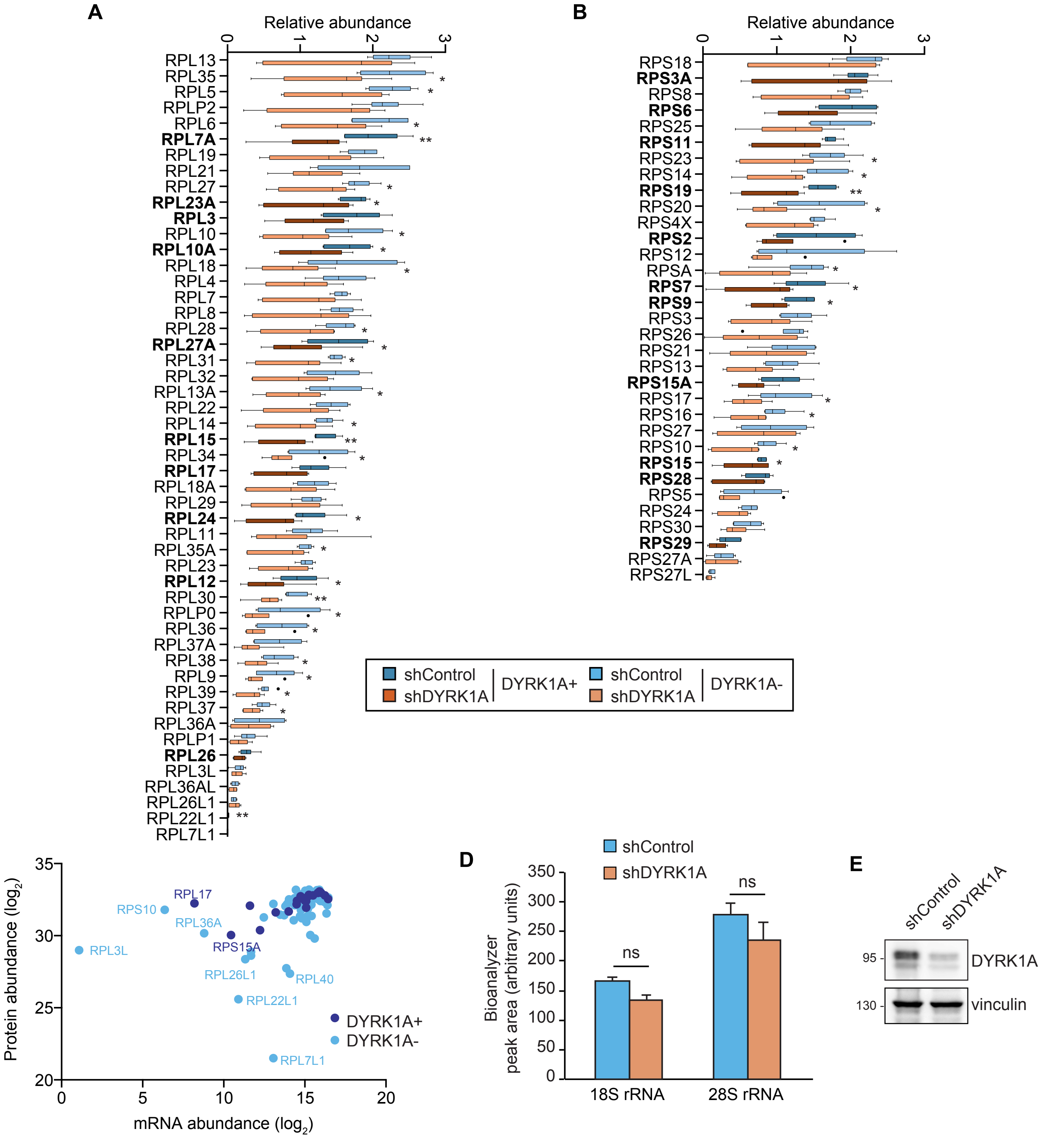

Figure S9. RP expression analysis based on MS quantitative data in T98G comparing shControl and shDYRK1A. (**A, B**) Relative abundance of RPs from the large (**A**) and small (**B**) ribosomal subunits in shControl and shDYRK1A T98G cells. The normalized level of each RP is shown relative to the mean of all RP values (n=6, for each condition; unpaired two-tailed Mann-Whitney test, **p*<0.05; ***p*<0.001). RPs whose encoding genes present DYRK1A at the promoter level are depicted in bold. (**C**) RPG mRNA levels determined by RNA-Seq are plotted relative to the normalized protein levels obtained by MS quantification. Both values are represented as log_2 values, and the blue and light blue dots correspond to RPGs bound by DYRK1A or not, respectively. (**D**) Quantification of 18S and 28S rRNAs was obtained by Bioanalyzer calculation of the areas of each of the peaks. The graph represents the mean ±SEM of 3 independent RNA samples for each condition (ns, not significant with an unpaired two-tailed Mann-Whitney test, yet differences for the 18S rRNA were significant when using the Student's test). (**E**) A representative image of the depletion levels of DYRK1A for samples used for MS identification shown in Figure 6 as assessed in WBs.

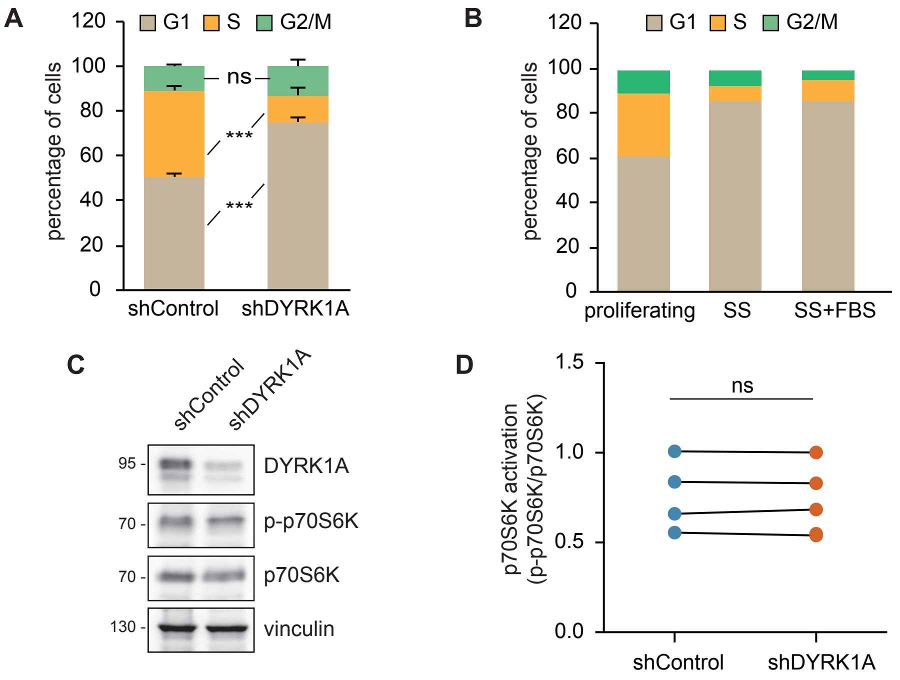

E

Experiment		shControl_1	shControl_2	shControl_3	shDYRK1A_1	shDYRK1A_2	shDYRK1A_3	tTest
Experiment 1	RPS6 total	6.34E+09	1.49E+10	4.40E+09	4.11E+09	3.50E+09	5.92E+09	
	[R].LS**S**^{236}LRA**S**^{240}TSK.[S]	6.23E+08	1.15E+09	3.94E+08	3.04E+08	3,09E+08	5.49E+08	
	Ratio phosphpeptide/total RPS6	9.82E-02	7.74E-02	8.96E-02	7.40E-02	8.82E-02	9.28E-02	7.25E-01
Experiment 2	RPS6 total	7.63E+09	2.25E+10	1.57E+10	1.13E+09	6.90E+08	1.26E+10	
	[R].LS**S**^{236}LRA**S**^{240}TSK.[S]	1.74E+07	1.43E+07	1.92E+07	1.52E+06	1.37E+06	1.67E+07	
	Ratio phosphpeptide/total RPS6	2.29E-03	6.34E-04	1.22E-03	1.35E-03	1.99E-03	1.33E-03	9.37E-01

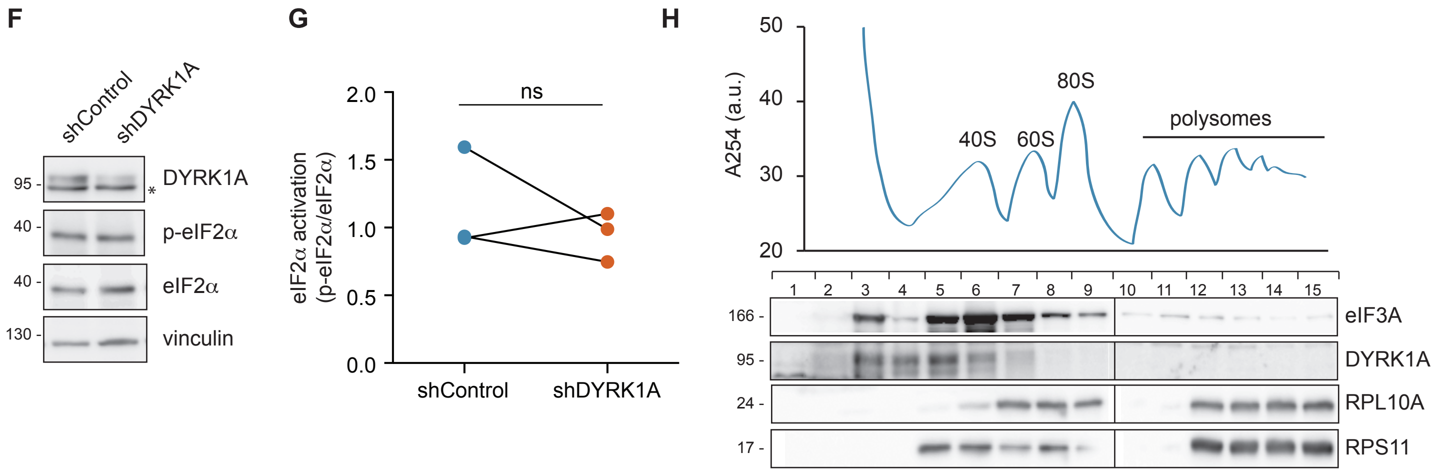

Figure S10. Supporting data for Figure 7. (**A**) Cell cycle profiles of T98G shControl or shDYRK1A infected cells determined by FACs analysis; the distribution of the G1, S and G2/M subpopulations is shown (mean ± SD, n=3 biological replicates, two-way Anova for multiple comparisons: ***$p \leq 0.001$, ns=not significant). Note that depletion of DYRK1A leads to an increase in the cells in G1 phase, concomitant with a reduction of cells in S phase. (**B**) Cell cycle profile of T98G cells grown under normal growth conditions (proliferating) and after serum starvation (SS) to show the increase in the G1 population. Re-addition of FBS (SS +FBS) did not induce changes in the cell cycle profile at 2 h after serum addition. (**C**, **D**) Analysis of Thr389 phosphorylation of p70S6K (p-p70S6K) by WB of shControl or shDYRK1A T98G cells. A representative experiment (**C**) and the quantification of the p-p70S6K signal relative to the total p70S6K (**D**) are shown (mean ± SD, n = 4, Wilcoxon matched-pairs signed ranks test, ns=not significant). The dots and lines show independent experiments. (**E**) TOP3 quantification of the RPS6 peptide containing phosphorylated Ser236 and Ser240 from shControl and shDYRK1A samples in MS experiments. RPS6 quantification in each sample is also shown, as well as the ratios between the two values. (**F, G**) Analysis of eIF2α Ser51 phosphorylation (p-eIF2α) in shControl or shDYRK1A T98G cells. A representative WB (**F**, *non-specific band) and the quantification of the phospho-eIF2α to total eIF2α ratio (**G**) is shown (mean ± SD, n =3: Wilcoxon test, ns = not significant). The dots and lines show independent experiments. (**H**) WB analysis of fractions from a polysome gradient to detect the presence of DYRK1A. Vertical lines indicate that the panel is not continuous. In the graph, the y-axis shows absorbance at 254 nm in arbitrary units and the *x*-axis corresponds to fractions with the top of the gradient to the left. A representative experiment of two performed is shown.
