# Supplementary material for: Loss of the DYRK1A Protein Kinase Results in the Reduction in Ribosomal Protein Gene Expression, Ribosome Mass and Reduced Translation
Source: Biomolecules. 2023 Dec 25;14(1):31. doi: 10.3390/biom14010031 (PMC10813206; doi:10.3390/biom14010031)
Supplement: Supplementary file 1 [file biomolecules-14-00031-s001.zip › 3_Tables S1-S4.pdf]

Table S1. Antibodies used in this study

Target	Host	Source	Working dilution
DYRK1A	Mouse	Santa Cruz (RR.7; sc-100376)	WB (1:1000); IP (2.5 μg)
DYRK1A	Rabbit	Abcam (ab69811)	ChIP (10 μg)
eIF2α	Rabbit	Cell Signaling (9722)	WB (1:1000)
eIF2α-p (pSer51)	Rabbit	Cell Signaling (9721)	WB (1:1000)
eiF3A	Rabbit	Bethyl (A302-002A)	WB (1:200)
RNA polymerase II (Rpb1)	Rabbit	Cell Signaling (14958)	ChIP (10 μg)
p70-S6K	Rabbit	Cell Signaling (9202)	WB (1:1000)
p70-S6K-p (pThr389)	Rabbit	Cell Signaling (9205)	WB (1:1000)
RPL10A	Mouse	Abnova (3G2; H00004736-M01)	WB (1:1000)
RPS11	Rabbit	Abcam (ab175213)	WB (1:1000)
α-Tubulin	Mouse	Sigma (DM1A; T6199)	WB (1:10000)
Vinculin	Mouse	Sigma (hVIN-1; V9131)	WB (1:10000)
ZBTB33/KAISO	Mouse	Santa Cruz (6F8; sc-23871)	ChIP (10 μg)
Anti-Mouse-HRP	Rabbit	Dako (P0260)	WB (1:2000-10000)
Anti-Rabbit-HRP	Goat	Dako (P0448)	WB (1:2000)

ChIP: Chromatin immunoprecipitation; IP: immunoprecipitation; WB: Western blot

Table S2. ENCODE datasets used in the analysis of chromatin occupancy

Factor	Cell Line	Sample	Dataset
GABPA*	SK-N-SH	GSM1010739	GSE32465
GABPA*	HeLa	GSM803454	GSE32465
MYC	GM12868	GSM822290	GSE33213
SP1	GM12868	GSM803363	GSE32465
TAF1	K562	ENCFF101GBL	ENCSR000BKS
TBP	HeLa	GSM935606	GSE31477
TBP	GM12868	GSM935277	GSE31477
TBP	K562	GSM935495	GSE31477
TBPL1	K562	ENCFF167PVP	ENCSR783EPA
YY1	GM12868	GSM803406	GSE32465
YY1	SK-N-SH	GSM1010897	GSE32465
ZBED1	K562	ENCFF465PNK	ENCSR286PCG
ZNF281	HepG2	ENCFF948PYK	ENCSR403MJY

*, GABPA data has been used as a proxy for GABP chromatin occupancy

Table S3. Primers for ChIP-qPCR

Gene	Forward primer	Reverse primer
RPS2	CGAGACCTACTGGGAAGCAG	GGAGCCAATCACACAGGTTGTC
RPS3A	ATAACAGGGCAAAGGTCACG	TTTCGTAAGGCGCTTGTTCT
RPS5	GCTGACCCGGAAGTTTTCTT	CACCTGAGAACACAGCCTGA
RPS6	CATCTTGAAGCAGCTGAACCG	CTCACTTCCGCTATCCCGTA
RPS7	GTTTCCGCCTCTTGTTC	AAAAGTTCTGTCGGAGAGCAG
RPS9	AAACAGAGAGGGTGGTTGA	CCACAACTACGCCAAAACCT
RPS11	GCTGAAGGCTGGTCACATCT	GGGCACTGTGAAGGACTGAC
RPS15A	GGAGAGCGCACGGAGTTAT	CCTTTCCCTCACCCTCGT
RPS19	AACTTTCGCCCTGAGAGAGG	CAGGGGAAAGGGAACGAC
RPL4	CCTTTTTGCGGAATAATCCAT	TCTTCAACTTCCGTCTGCAA
RPL7A	CCGCCTCGATTTTTAGCTTT	CGGGAGGAGAGAGAAAGGAA
RPL10	GCAAGCTCAGGGACACTCTC	CTTGGGCGTAGAACTCTTGC
RPL10A	CAACCGCTCTGCGGGTAG	GCGCTAACCGGAAAAGAGAC
RPL12	GCGGACAAGCCAGATATAGG	CTGCCCACAACAAACATGG
RPL17	AGGCTGCTTAGGGAAAGAGG	TGCCTCCTCAGATTCGTTT
RPL18	GGGCGTTTCCTTATCAGGTT	GATCATCTCGGGTTAGAGCCTT
RPL23	ACCAGGGCCTAATCCAGTCT	GGAGAGTTGAGATGGCAGGA
RPL26	CCGCAAAAGGGAAGAGAACT	CAGTTTACTCCCCTCGCTCA
RPL27A	GGATCACCACCCTCAGAAAG	CCTCACTTCCGGTCACAGAG

Table S4. Primers for RT-qPCR

Gene	Forward primer	Reverse primer
Act42a[a]	GCGTCGGTCAATTCAATCTT	AAGCTGCAACCTCTTCGTCA
DYRK1A	CCTTGATAGGCAAAGGTTCC	CGCACTTCTATCTGTGCTTG
EIF4E2	ATGATGACAGTGGGGACCAT	GTTGTACTGCAGGGGATGCT
RPS2	AAGATCAAGTCCCTGGAGGA	TGCTTCTGCACTGGCATAAT
RPS6	AGAAGATGATGTCCGCCAGT	CTGCAGGACACGTGGAGTAA
RPS15A	AACCTCACAGGCAGGCTAAA	CGGGATGGAAGCAGATTATT
RPS19	CAAAGAGCTTGCTCCCTACG	TTCTCTGACGTCCCCCATAG
RPS24	CGTGCGCGTTGATATGATT	GCGGATAGTTACGGTGTCGT
RPL7	GGAACCATGGAGGGTGTAGA	TTTCTCAGGCGCTTGATCTT
RPL7A	AGAAGGCCAAGGGAAAGAAG	AGGTCTCTTTTGGGCTGGAT
RPL14	GTGCATGCAGCTCACTGATT	TTCAATCTTCTTGGCCCATC
RPL17	GCTGCACATGCTTAAAAACG	GCGCATCTTAGGTGCTTTGT
RPL21	AGTTGTTCCTTTGGCCACATA	GGTAACACTTGTGGGGCATT

RPL26	GGAAAAGGCTAATGGCACAA	TCCTTTCCTACTTGGCGAGA

[a], primers targeting *Act42a* from *D. melanogaster*
